# Simulation of oak early life history and interactions with disturbance via an individual-based model, SOEL

**DOI:** 10.1371/journal.pone.0179643

**Published:** 2017-06-20

**Authors:** Kenneth F. Kellner, Robert K. Swihart

**Affiliations:** Department of Forestry and Natural Resources, Purdue University, West Lafayette, IN, United States of America; University of Missouri Kansas City, UNITED STATES

## Abstract

Early tree life history and demography are driven by interactions with the environment such as seed predation, herbivory, light availability, and drought. For oak (*Quercus*) in the eastern United States, these interactions may contribute to oak regeneration failure. Numerous studies have examined the impact of individual factors (like seed predation) on the oak regeneration process, but less information is available on the relative and combined impacts of multiple intrinsic and extrinsic factors on early oak life history. We developed an individual-based, spatially explicit model to Simulate Oak Early Life history (SOEL). The model connects acorn survival, acorn dispersal, germination, seedling growth, and seedling survival submodels based on empirical data with an existing gap model (JABOWA). Using SOEL, we assessed the sensitivity of several metrics of oak regeneration to different parameters associated with early oak life history. We also applied the model in three individual case studies to assess: (1) how variable acorn production interacts with timing of timber harvest; (2) the effect of shelterwood harvest-induced differences on seed predation; and (3) the consequences of interactions between drought, seedling growth and survival, and timber harvest. We found that oak regeneration metrics including percent emergence, seedling density, and sapling density were most sensitive to the amount of acorn production, acorn caching probability by scatterhoarders, and seedling growth rates. In the case studies, we found that timing harvest to follow large acorn crops can increase the density of oak regeneration in the short term following harvest, at least under some conditions. Following midstory removal, lower weevil infestation probability and lower post-dispersal acorn survival resulted in a modest decline in seedling density, but the decline did not persist to the sapling life stage class. Drought frequency had a powerful negative impact on both growth and survival for individual seedlings, which resulted in large reductions in both seedling and sapling density. The case studies presented here represent only a few examples of what could be accomplished within the SOEL modeling framework. Further studies could focus on different early life history parameters, or connect the parameter values to different predictor variables based on field data.

## Introduction

Early tree life history and demography is driven by both intrinsically and extrinsically mediated interactions with the environment. The relative importance of intrinsic or extrinsic factors falls on a continuum varying with time, space, and the ecological process in question. For example, seed production is primarily an intrinsic process, but weather can also influence seed crops [1]. In contrast, seed predation is primarily an extrinsic, biotic interaction driven by predators, but seed traits (as determined by the parent tree) can play a role in the predation and dispersal process [2]. Seedling growth and survival are also typified by substantial impacts of extrinsic, biotic factors including herbivory [3,4] but also abiotic environmental conditions including light and drought [5]. Together, these early life history processes drive the abundance and composition of tree seedlings in the forest understory and influence the future successional path of the forest [6]. Tree early life history is therefore important for understanding forest ecology and informing forest management to shape successional trajectories. Thus, understanding the interactions between management and the intrinsic and extrinsic factors that influence early tree life history is an important research goal [7].

Oaks (*Quercus*) are a frequent research subject of forest ecologists and a management focus for foresters [8,9]. They are a dominant component of the canopy in many eastern hardwood forests, a key food source for numerous species of mammals and birds, and a valuable timber species [9,10]. The decline of oak throughout eastern forests over the past century, likely a consequence of changing patterns of forest disturbance [11], has prompted numerous studies on oak ecology and management for oak regeneration [9]. Successful oak management requires the presence of advance regeneration; that is, young oak stems already in the understory, originating from seed or sprout, ready to capture the increased light and newly created growing space [8].

Seed-origin regeneration in early oak life history involves (1) acorn production, (2) acorn predation and dispersal, and (3) seedling growth and survival. To the detriment of managers, these processes are frequently unpredictable, correlated with each other, and/or altered by disturbances associated with forest management. Acorn production, for example, is highly variable year-to-year and spatially autocorrelated within a given oak species [1]. The overall effect of harvest on acorn predation is unclear, but any effect is likely to be small compared to yearly variability [12–15]. The cycle of acorn production induces correlated cyclical patterns in acorn predators including acorn weevils (*Curculio* and *Conotrachelus*) and granivorous small mammals; rates of infestation and predation are typically maximized when acorns are scarce [15–18]. Forest management alters habitat structure for acorn predators and thus can potentially impact the infestation, predation, and dispersal processes by changing seed predator abundance and/or behavior [13,15,19,20].

Seedling growth and survival are driven by their own suite of extrinsic factors. Available light—usually a direct effect of forest management—is among the most important predictors of seedling growth given oak’s relative intolerance to shade [21]. However, an increase in available light following harvest can also promote faster-growing oak competitors (e.g. tulip poplar, *Liriodendron tulipifera*) and thus have an indirect negative impact on oak [22,23]. Additionally, under drought conditions, seedlings in high-light environments may suffer greater physiological consequences than seedlings in shade [24,25]. Biotic factors, namely herbivory by insects and mammals on oak seedlings, can also limit establishment [26–28]. As with acorn production and predation, forest management can alter the abundance and habitat use of herbivores and thus impact the level of herbivory on oak seedlings [29–31].

Intrinsic and extrinsic factors that affect acorn production, predation, and oak seedling growth and survival likely contribute to variable success when managing for oak regeneration and has motivated substantial research on early oak life history and its interaction with management [8,32]. Previous field studies generally have focused on a single process (e.g., seed production) or a single oak life history stage (e.g., seedling) [13–15,20,33]. A narrow focus is helpful in understanding the process being studied, but it precludes direct assessment of the relative importance of different component processes (e.g. seed production, predation, herbivory) on advance oak reproduction under different management regimes. Demographic modeling permits simultaneous examination of multiple processes and life stages. Approaches for trees include matrix models [34], integral projection models [35], and individual-based models [36]. Among these are oak-specific ACORn [37] and SIMSEED [38], which focus on simulating advance reproduction. SIMSEED allows for variability in oak seedling recruitment and survival [38], but neither framework explicitly models intrinsic and extrinsic factors impacting these vital rates.

To simultaneously examine intrinsic and extrinsic drivers of early oak life history and interactions with forest management, we developed a new individual-based demographic model (hereafter SOEL, or Simulation of Oak Early Life history) for black (*Q*. *velutina*) and white (*Q*. *alba*) oaks that included acorn production, acorn predation and dispersal, and seedling growth and survival components. Importantly, oaks in SOEL were placed in the context of a spatially explicit simulated forest, including competitor species and spatially variable light availability. The modeling framework allowed us to run experiments via simulation that would be difficult to conduct in the field and track the impact of changes in individual life history parameters on the density and size distribution of oak regeneration. We performed global sensitivity analysis on the model to determine the relative importance of key early life history parameters (Table 1) on oak demography. We then applied SOEL to three case studies involving the interaction of early oak life history and timber harvest, based on findings from our previous field studies [15,20,31,39]. First, we examined how variable acorn production interacts with timing of timber harvest. Second, we examined the effect of shelterwood harvest-induced differences in seed predation. Finally, we examined the consequences of interactions between drought, seedling growth and survival, and timber harvest.

**Table 1:** Key oak early life history parameters.

Parameters		Empirical Data		Fitted Regression Model			
Name	Description	Source	Years	TE^a^	YE-Mast^a^	YE-Random^b^	Other Covariates
*meanAcorn*	Mean acorns produced per m^2^ canopy area	[15]	9	0	0	Yes	N/A
*pWeevil*	Probability of acorn weevil infestation	[15]	9	-	-	Yes	N/A
*pDispersal*	Probability of acorn dispersal	[15]	9	+	0	Yes	Species
*dispDist*	Weibull scale parameter for dispersal kernel	[20]	4	-	-	No	N/A
*pCache*	Probability dispersed acorn is cached	[20]	4	0	0	Yes	Distance dispersed
*pDispEaten*	Probability acorn eaten | dispersed	[20]	4	+	-	No	Species, Cached status
*pUndispEaten*	Probability acorn eaten | not dispersed	[20]	4	+	-	No	Species
*pGerm*	Probability of acorn germination	[40,41]	N/A	N/A	N/A	N/A	Weevil status, cached status
*pBrowse*	Probability of browse damage on seedling	[31]	4	0	0	Yes	Species, Height, Height^2^
*meanGr*	Yearly seedling growth	[39]	4	0	0	No	Light, Species, Browsed
*pSurv*	Yearly seedling survival	[39]	4	0	0	No	Light, Species, Age

Empirical data for all parameters except *pGerm* were collected from studies at the Hardwood Ecosystem Experiment (HEE) [43]. Regression models were then fit to each parameter, with a pool of potential predictors including midstory removal treatment effect (TE), yearly effect of mast availability (YE-mast), and random effect of year (YE-Random) selected for inclusion based on significance (*p* < 0.05). Detailed information on the regression models can be found in S2 Code.

^a^The relationship of each parameter with TE and YE-mast is indicated with + (positive relationship),—(negative), or 0 (no effect / not included in model)

^b^“Yes” indicates a random effect of year was included in the fitted model; “No” if not.

## Materials and methods

### Location

Empirical data to parameterize SOEL were collected at Morgan-Monroe and Yellowwood State Forests in southern Indiana, USA (39°06’–39°21’ N, 86°16’–86°27’ W) with permission of the Indiana Department of Natural Resources, Division of Forestry. Together the state forests cover > 18,000 ha of mainly upland areas with steep slopes [22]. The forest canopy is dominated by hardwoods, primarily oaks (*Q*. *alba*, *Q*. *velutina*, *Q*. *prinus* and *Q*. *rubra*) and hickories (*Carya*), and the understory is mainly shade tolerant maple (*Acer*) and beech (*Fagus grandifolia*) [42]. The primary acorn predators are small mammals, including white footed mice (*Peromyscus leucopus*), eastern chipmunks (*Tamias striatus*) and gray squirrels (*Sciurus carolinensis*), and the primary herbivores are white-tailed deer (*Odocoileus virginianus*) and eastern cottontail rabbits (*Sylvilagus floridanus*) [20,31]. Data collection was centered on long-term monitoring sites established as part of the Hardwood Ecosystem Experiment (HEE), a long-term study of the effects of silviculture on forest ecosystems [43].

### Demographic data

Data on acorn production, acorn predation and dispersal, and seedling survival and growth for black and white oak were obtained from multiple studies conducted at the HEE between 2006 and 2014. All studies were replicated across multiple sites and management treatments (no harvest, clearcut harvest, and the first, midstory-removal phase, of a 3-phase shelterwood harvest) which were implemented in 2008–2009 [43]. The midstory removal phase removed non-oaks <25.4 cm d.b.h. down to a minimum residual basal area of 13.8 m^2^/ha, and the clearcut removed all stems > 1 cm d.b.h. in a single harvest event [43].

Yearly acorn production data (in acorns per m^2^ of canopy) were collected from 56 white and 57 black oaks over the 9-year period (64 in unharvested areas, 31 on the edge of harvest openings, and 18 in midstory removal areas) [15]. Collected acorns were X-rayed to determine weevil infestation status and calculate a yearly infestation probability. Under each study tree, fallen acorns were marked in the fall and monitored for removal by seed predators to calculate a dispersal probability. For a subset of 4 years (2011–2014) acorns at 10 trees (5 each in unharvested and midstory removal sites) were tagged and then relocated using metal detectors the following spring to determine acorn caching and survival probabilities [20].

To obtain data on oak seedling survival and growth, 39 black and white oak seedlings (24 first-year seedlings and 15 year-old seedlings) were experimentally planted in each of 27 plots located within unharvested control (n = 6), clearcut (n = 18), and midstory removal sites (n = 3) in May 2011 [31,39]. Seedlings were re-visited twice yearly through 2014 to assess survival, measure growth and light availability, and identify herbivore damage and insect defoliation.

### Modeling approach

We modeled oak demography with SOEL using a multiple-step process [44]. First, we developed individual regression models for early oak life history parameters using empirical data and generalized linear mixed modeling techniques, and organized these parameters into an “early life history” submodel tracking individual acorns from source tree to seedling (defined as < 1.4 m height). Second, we developed a “contextual forest” submodel adapted from the established JABOWA-II (hereafter JABOWA) forest modeling framework to simulate vital rates of saplings (≥ 1.4 m height, < 1.5 cm d.b.h.) and mature trees (≥ 1.5 cm d.b.h) [45]. We selected JABOWA for three reasons: first, a detailed model description was available and could be adapted to fit our modeling framework [45]. Second, JABOWA has been widely tested, applied and modified [36]. Finally, JABOWA requires relatively few model parameters, which were readily available in the literature (a requirement as we did not have empirical data on trees larger than saplings) [45]. Finally, we linked the two submodels to form a comprehensive forest model focused on oak. An overview of the two submodels is provided below; for a detailed description of the model and the source code see provided supporting information (S1 Appendix and S1 Code).

**Fig 1:**
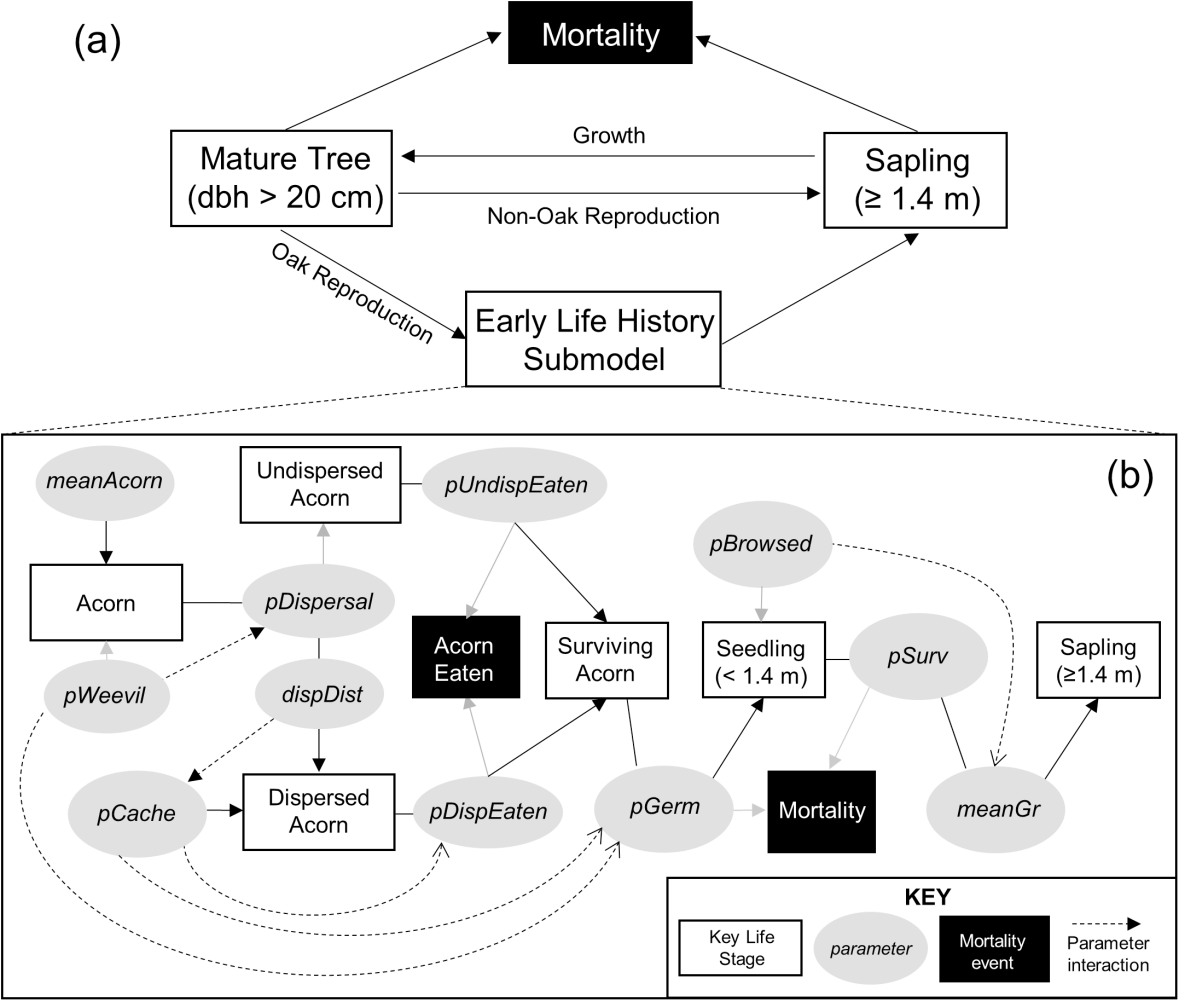
SOEL model structure. SOEL is composed of (a) the contextual forest submodel and (b) the early life history submodel. Parameter descriptions are found in Table 1.

The early oak life history submodel was governed by 11 key oak early life history parameters, of which 8 were related to the acorn life stage and the remaining 3 to the seedling life stage (Table 1, Fig 1). For the 10 parameters for which we had empirical data, we fit unique generalized linear mixed models in R and JAGS [46,47]. For each regression, we defined a saturated model of candidate predictor variables based on prior analyses of the empirical data [15,20,31,39]. For acorn-related parameters, oak species, midstory removal effect, yearly ambient mast availability, and yearly random effects were included in the candidate set at a minimum; for seedling-level parameters, species, age, height and effect of light availability were included. Ultimately, only significant predictor variables were retained in the final model for each early life history parameter (Table 1). Detailed information about the fitted models can be found in S2 Code.

The contextual forest submodel simulated the spatially explicit forest structure contextualizing the early oak life history submodel, comprising trees ≥ 1.4 m in height (Fig 1). Four tree species were included: (1) black and white oaks (which have passed through the early life history submodel and reached ≥1.4 m height); (2) a shade-tolerant oak competitor (sugar maple, *Acer saccharum*); and (3) a shade-intolerant oak competitor (tulip poplar). Since we lacked empirical data for saplings or mature trees, we used an established modeling framework (JABOWA) to simulate their yearly growth and survival due to its ease of parameterization from the literature [45,48]. Briefly, JABOWA simulates tree diameter growth as a species-specific function of current tree diameter, height, light availability, a site quality index and stand basal area [45]. Trees shade nearby shorter trees, and light availability at any given spatial location and height (a necessary input for the early life history submodel) is calculated based on the depth of the canopy above [45]. Yearly tree survival is a function of tree age relative to maximum age, with a penalty to survival probability if diameter growth is below a threshold level of 0.01 cm [45]. For the two non-oak species, JABOWA was also used to simulate regeneration; in JABOWA new saplings of a given species are added to the simulation with a probability based on site quality and light availability [45].

JABOWA is only partially spatially explicit. Individual trees are located indistinctly within 10x10 m cells, and the canopies of all trees in a cell are assumed to fill the entire cell area. To match the more spatially explicit early life history submodel, we modified several aspects of JABOWA. First, all trees were given unique spatial coordinates. Second, canopy dimensions (and thus patterns of shade cast) were circular and related to tree height based on allometric equations. Third, the negative effect of stand basal area on individual tree diameter growth [45] was replaced with a negative effect of nearby stem density (in a 3.5 m radius) which facilitated a more realistic forest structure and accumulation of basal area (S1 Appendix). Finally, trees were allowed to sprout directly into new saplings following mortality events based on their diameter, using species-specific predictive sprouting models from the literature (e.g. [49]).

The two submodels were linked together in the NetLogo individual-based modeling framework [50] to form the complete forest model (Fig 1). In all simulations, forests were initialized at a size of 140 x 140 m (roughly 2 ha) with a tree composition matching the size and species distribution of forest structure data collected at the HEE [42]. In each annual model time step, acorn production, acorn dispersal and survival, and seedling growth and survival were simulated stochastically using parameter values generated by the set of predictive regression equations (Table 1).

All simulations were run for 37 time steps (i.e., years): a 30-year spin up period followed by a 7-year data collection period. The 30-year spin up period was included to minimize the impact of the initial forest composition and configuration and allow the simulated forest to reach a stable state. Seven years was chosen because it corresponded to the number of years of post-harvest empirical data collected at the HEE. Multiple output parameters related to oak regeneration were recorded following each time step in this period: (1) the density of oak seedlings per ha at the beginning of the 7-year period (hereafter SEEDDENS); (2) the total number of acorns produced per ha over the 7-year period (TOTAC); (3) the mean percent of produced acorns that ultimately emerged as seedlings over the 7-year period (PCTEMR); (4) the total number of new seedlings that entered the simulation during the 7-year period per ha (< 1.4 m height; TNSEED), and (5) the density of oak saplings at the end of the 7-year period per ha (defined as ≥ 1.4 m height and < 1.5 cm d.b.h; SAPDENS). The model was called from within R using package RNetLogo [51]. Complete code for SOEL and the R script used to control simulations are provided as supporting information (S1 Code).

### Validation

We performed model validation by comparing output from SOEL with empirical data from the HEE as well as simulation output from unmodified JABOWA. Using default (i.e., mean) input parameters, we conducted 30 replicate simulations in both SOEL and an implementation of JABOWA in NetLogo (S1 Code). Each simulation was composed of a 30-year spin-up period followed by a harvest treatment (clearcut, shelterwood, or no harvest). For each SOEL simulation replicate, we recorded the total number of acorns produced and the average number of acorns produced per mature oak in the 10 years prior to harvest. We compared this distribution of acorn production with empirical estimates from the HEE data and from the literature to determine if SOEL was generating ecologically realistic predictions of seed production.

In each replicate SOEL simulation, we also recorded the density of oak seedlings and saplings five years following application of the harvest treatment. In the JABOWA simulations, we recorded only sapling density (since the seedling stage is not simulated). We compared these values with empirical estimates of oak seedling (<1.4 m height) and sapling (≥ 1.4 m, < 1.5 cm dbh) density obtained across the HEE sites five years following each harvest treatment. Seedling and sapling densities were calculated from 16 m^2^ vegetation survey quadrats located in each harvest treatment (*n* = 31 in clearcuts; *n* = 21 in shelterwoods; *n* = 433 in unharvested areas).

Finally, we tested the ability of SOEL to simulate realistic stand development (following a stand initiation / disturbance event) that could result in a forest approximating the structure of the forest stands at the HEE. For both SOEL and JABOWA, we conducted 30 replicate simulations that began with a stand initiation event (a clearcut harvest). In each simulation year post-clearcut, we recorded several key metrics of forest structure (basal area, stem density, and quadratic mean diameter) and compared these values with the range of empirical data on mature forest structure collected at the HEE [42].

### Global sensitivity analysis

We conducted a global sensitivity analysis for the subset of early oak life history parameters in SOEL for which we had empirical data (Table 1). In a global sensitivity analysis, all parameters of interest are simultaneously perturbed within a given distribution, and the impact of this variability on a given model output is determined [44]. The input distribution for each parameter was normal, with a mean equal to the estimate of the regression model intercept for the parameter (S2 Code); the standard deviation was equal to the standard deviation around the estimate, representing the level of uncertainty in the parameter mean. The analysis was performed using a Monte Carlo approach: 500 sets of input parameters were drawn from the appropriate distributions in a Latin hypercube design while explicitly accounting for correlation between the parameters [52,53]. For each set of input parameter values, we ran a single 37-year simulation (30 year spin-up + 7 year data collection period). Sensitivities of three output metrics (PCTEMR, TNSEED, SAPDENS) to each input parameter were then calculated by separating the uncorrelated component of sensitivity for a parameter with the component correlated with the other input parameters [53].

### Case study: Interaction of harvest timing and acorn production

To maximize the amount of oak regeneration that can benefit from the release of a harvest event, managers often attempt to time harvests (particularly shelterwood cuts) to follow years of high acorn production and the resulting abundance of oak seedlings [9]. This approach can be challenging given the unpredictability of year-to-year acorn production [1], and few studies have attempted to empirically quantify the extent to which acorn crops of different sizes translate into densities of oak seedlings and saplings. By manipulating acorn production (parameter *meanAcorn*) prior to harvest events, SOEL can provide simulated estimates of these metrics.

In this case study, we assessed the effects of timing harvests to coincide with high acorn production via four acorn production “bumper crop” scenarios: (1) *meanAcorn* 1 standard deviation (SD) above the mean value for 1 year prior to harvest; (2) 1 SD above the mean for 2 years prior; (3) 2 SD above the mean for 1 year prior; and (4) 2 SD above the mean for 2 years prior. Acorn production mean and SD were defined based on the corresponding fitted regression model for *meanAcorn* with yearly random effects (Table 1, S2 Code). In all other simulation years, and in the entirety of an additional average scenario, *meanAcorn* was allowed to vary randomly based on the regression model. We crossed the five total acorn production scenarios with three harvest treatments applied to the simulated forest following the 30-year spin-up: no harvest, a midstory removal, and a clearcut harvest. Each scenario × harvest treatment combination was applied to 30 replicate simulations with other parameters held constant at average values. Two oak regeneration output metrics (SEEDDENS, SAPDENS) were recorded in each simulation.

### Case study: Harvest and yearly variation in seed predation processes

Seed production, and thus ambient food availability, has been shown to influence the seed predation and dispersal process [54]. When many seeds are produced, a given seed is more likely to escape predation and germinate successfully; this has been called the “predator satiation hypothesis” and is one potential explanation for the masting behavior of many tree species including oaks [54]. At the HEE, high variability in predation by weevils and small mammals was likely due in part to fluctuations in acorn production [15,20]. Changes in habitat structure may also affect seed predation. For example, we found evidence of a decrease in predation by acorn weevils and an increase in post-dispersal predation of acorns by small mammals following midstory removal as part of a shelterwood harvest [15,20]. While interesting from an ecological perspective, the ultimate impact on oak regeneration of yearly and harvest-induced variation in seed predation is unclear.

For this SOEL case study, we constructed regression models for seed predation and dispersal parameters (*pWeevil*, *pDispersal*, *dispDist*, *pCache*, *pDispEaten*, and *pDispUneaten*) that allowed for yearly variability and effects of midstory removal (Table 1, S2 Code). By running simulations that incorporated one or both of these effects and comparing them to simulations in which the parameters were held constant, we were able to measure the impacts on output metrics of oak regeneration. We defined four scenarios: (1) a control scenario in which there was no yearly variability or harvest effects; (2) harvest treatment effects but no yearly effects; (3) yearly effects but no harvest effects; and (4) both harvest and yearly effects. We crossed these four scenarios with two harvest treatments: no harvest or midstory removal. Note that some scenarios in the no harvest treatment had parameters models that included a potential harvest treatment effect, but had no harvest take place, serving as secondary controls. As with the previous case study, all scenario × harvest treatment combinations were replicated 30 times each with harvests (if applicable) occurring immediately after the 30-year spin-up period. Values for four output metrics (TOTAC, PCTEMR, TNSEED, SAPDENS) were recorded for each replicate during the subsequent 7-year period.

### Case study: Interactions of drought and harvest

Oak seedlings are relatively drought-tolerant compared to competitors, thanks in part to morphological adaptations like a well-developed taproot [55]. However, drought conditions may exceed this tolerance with consequences for seedling survival and growth [56,57]. Climate change is projected to increase the frequency of extreme weather events including drought in the Midwestern United States [58]. Thus, understanding the impact of drought on the accumulation of oak regeneration is an important research goal.

At the HEE, we monitored oak seedling growth and survival over a 4-year period which included two drought years [39]. The two drought years were defined by very low precipitation during the growing season (total July precipitation was 16.3 and 14.2 mm, respectively, compared to a 30-year average of 109 mm) [59]. The entire study area reached at least “severe drought” (category D2) status for at least part of the growing season in both years, according to the U.S Drought Monitor [60]. The drought reduced seedling growth and survival, and the effects were greatest in clearcut harvests where seedlings were most exposed to desiccation [39].

To simulate the impact of a drought year on seedling growth and survival in SOEL, we fit separate regression models for seedling growth and survival (parameters *meanGr* and *pSurv*) to the data for the drought and non-drought periods (Table 1, S2 Code). Then, we defined a probability parameter within the simulation that represented the chance that a given simulation year was a drought year or not, applying the appropriate predictive equations for seedling growth and survival. We defined six drought scenarios in which drought probability ranged from 0 to 1 in increments of 0.2. These scenarios were crossed with three harvest treatments: no harvest, midstory removal, and a clearcut harvest. As with previous case studies, each drought scenario × harvest treatment combination was replicated 30 times with each replicate consisting of a 30-year spin-up period, followed by harvest treatment (if applicable), followed by a 7-year data collection period for two metrics (TNSEED and SAPDENS).

### Analysis of model output

For the acorn production and seed predation case studies described above, we examined the interactive effects of harvest treatment and scenario on each metric of oak regeneration using analysis of variance in R [46]. Post-hoc differences between groups (defined as scenario × harvest treatment) were assessed with Tukey’s honest significance test. For the drought case study, scenario (i.e., drought probability) was a continuous variable. Thus, we examined the interaction of harvest and drought probability on oak regeneration metrics using linear regression.

## Results

### Validation

Mean acorns year^-1^ tree^-1^ for mature trees (dbh > 15.2 cm) from the model was 922 with a standard error of 209, which fell in the middle of the range reported in the literature (382–2147; Table 2). At the stand scale, a mean of 90,757 acorns year^-1^ hectare^-1^ were produced by the simulation model. This value was similar to values reported by other studies in oak-dominated forests (Table 2), with the important qualification that acorn production is dependent on the basal area and size distribution of oak within the stand.

**Table 2:** Acorn production validation.

				Acorns tree^-1^ year^-1^		Acorns ha^-1^ year^-1^		
Source	U.S. State	Species	Years	Mean	SE	Mean	Min	Max
SOEL	N/A	RO, WO	10	922	209	90,757	6029	193,481
[61]	MI	WO	6	1100	400	N/A	N/A	N/A
[62]	MI	RO	8	1100	368	N/A	N/A	N/A
[62]	MI	RO	8	714	295	N/A	N/A	N/A
[63]	NC	RO	10	680	266	N/A	N/A	N/A
[63]	NC	WO	10	2595	1017	N/A	N/A	N/A
[15]	IN	RO	6	424	137	N/A	N/A	N/A
[15]	IN	WO	6	382	147	N/A	N/A	N/A
[64]	MN	RO	1	N/A	N/A	151,000	N/A	N/A
[65]	PA	RO	4	N/A	N/A	103,236	1300	490,518
[66]	IL	RO, WO	1	N/A	N/A	212,619	N/A	N/A
[67]	IN	RO, WO	1	N/A	N/A	180,214	N/A	N/A
[68]	MO	RO, WO	15	N/A	N/A	N/A	<10,000	180,000

SOEL-derived metrics of acorn production by mature oak trees (dbh >15.2 cm) based on 10 simulated years, compared to production estimates in the literature from species in the red oak section (RO) and the white oak section (WO) in several U.S. states.

**Fig 2:**
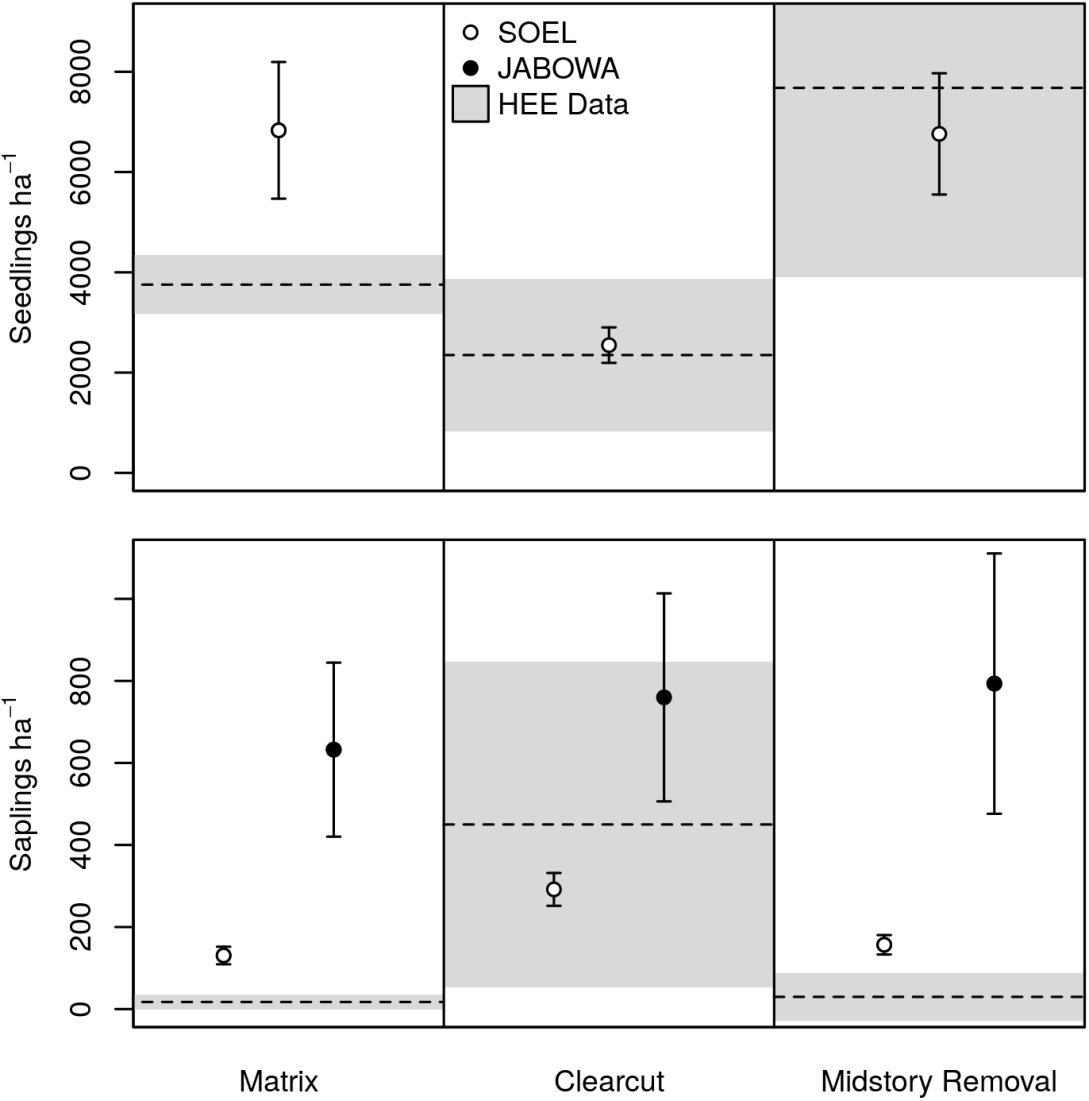
Oak seedling density validation. Predicted densities of oak seedlings (<1.4 m height) from SOEL and saplings (≥ 1.4 m and <1.5 cm DBH) from SOEL and JABOWA under three harvesting scenarios were compared to observed densities from the Hardwood Ecosystem Experiment (HEE). Model results are means and 95% confidence intervals from 30 replicated simulations (measured 5 years postharvest to match HEE data). The dashed line represents the mean from the HEE field data and the shaded area is the 95% confidence interval around the mean.

SOEL generated reasonable predictions of oak seedling and sapling density (Fig 2). Both seedling and sapling densities were slightly overestimated under the no harvest treatment. In contrast, JABOWA predicted much higher oak sapling densities (particularly for the no harvest and shelterwood treatments) than were observed (Fig 2). The contrast in sapling density between SOEL (similar to the empirical data) and JABOWA suggests that the simplistic regeneration process used in the latter ignores the challenges facing oak early in its life history (weevil infestation, predation, herbivory) thus overestimating the number of oaks that reach the sapling stage.

**Fig 3:**
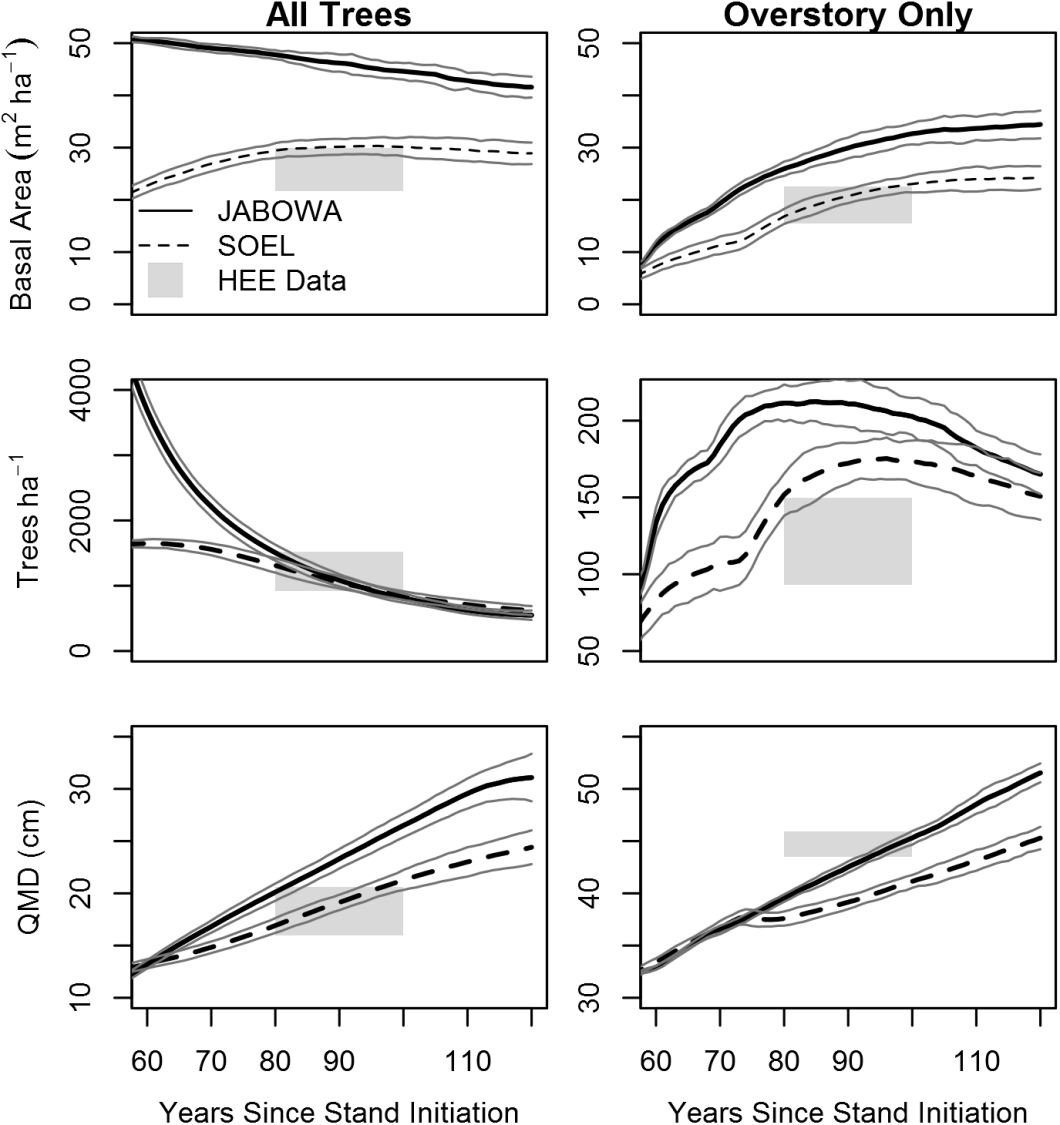
Forest structure validation. Three metrics of forest structure (basal area, stem density, and quadratic mean diameter) were compared between forest stands simulated by JABOWA and SOEL (means of 30 replicates) and empirical data gathered at the HEE sites. Comparisons are for all trees (dbh >1.5 cm) and overstory trees only (dbh >30 cm). HEE data represents the range of values reported in 9 forest stands approximately 80–100 years old. Gray lines represent one standard deviation above and below the simulation mean.

Simulated forests 80–100 years post-disturbance generally had similar structure to the HEE stands, particularly when all stems > 1.5 cm dbh were considered (Fig 3). When only overstory trees (dbh > 30 cm) were measured, the model slightly overestimated density with a corresponding underestimate of quadratic mean diameter (Fig 3). Still, SOEL more accurately approximated forest structure than did JABOWA, particularly for stand basal area. A complicating factor is that the HEE forest stands used for this comparison have been subjected to some single-tree selection harvesting since stand initiation, likely reducing overall stand basal area, while the simulated stands were not harvested following the initial disturbance event.

### Sensitivity analysis

The global sensitivity analysis identified several parameters that had large influence on oak regeneration metrics. Metrics PCTEMR and TNSEED over the 7-year data collection period were sensitive to variability in acorn caching probability (*pCache*) (Table 3). SAPDENS was sensitive to caching probability, mean seedling growth (*meanGr*), and to a lesser extent, probability of seedling survival (*pSurv*) (Table 3). Both TNSEED and SAPDENS were sensitive to acorn production (*meanAcorn*), but a relatively small amount of this sensitivity was unique and uncorrelated with other parameters. A high level of correlation existed between *meanAcorn* and other acorn-level parameters, a relationship that was also reflected in the fitted models for several acorn-level parameters (Table 1).

**Table 3:** Sensitivity analysis.

	PCTEMR		TNSEED		SAPDENS	
Parameter	*S*	U	*S*	U	*S*	U
*meanAcorn*	0.068	0.00	0.419	0.20	0.305	0.15
*pWeevil*	0.026	0.00	0.044	0.06	0.010	0.16
*pDispersal*	0.047	0.20	0.005	1.36	0.025	0.27
*dispDist*	0.028	0.00	0.027	0.01	0.072	0.00
*pCache*	0.864	0.69	0.459	0.48	0.391	0.35
*pDispEaten*	0.134	0.00	0.146	0.00	0.200	0.00
*pUndispEaten*	0.121	0.01	0.082	0.00	0.140	0.00
*pBrowse*	0.001	0.10	0.052	0.00	0.029	0.03
*meanGr*	0.067	0.00	0.079	0.00	0.201	0.09
*pSurv*	0.118	0.08	0.057	0.12	0.170	0.23

Sensitivity (*S*) of three different metrics of oak regeneration to key early oak life history parameters in the individual-based model obtained using global sensitivity analysis [53]. The proportion of the sensitivity uncorrelated with other parameters is represented by U. Values of U larger than 1 are possible when input parameters are negatively correlated, resulting in a negative correlated contribution to sensitivity [53].

### Interaction of harvest timing and acorn production

**Fig 4:**
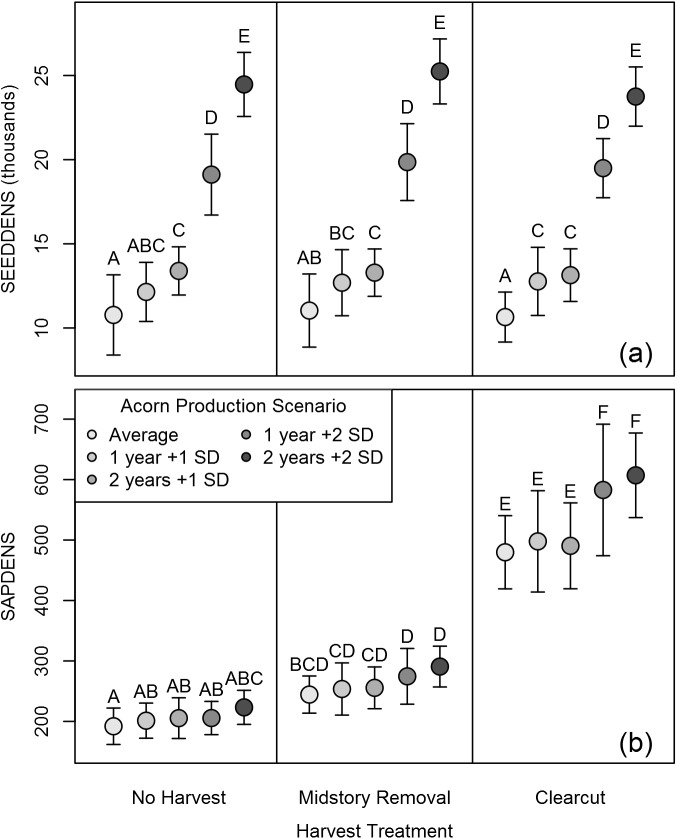
Effect of harvest timing on acorn production. Mean (± standard deviation) values for (a) SEEDDENS and (b) SAPDENS simulation output metrics for five different pre-harvest acorn production scenarios and three different timber harvest treatments. Acorn production scenarios were compared to a control/average scenario (random production prior to harvest) and were defined by crossing 2 variables: the magnitude of “good” acorn production (1 or 2 standard deviations above the average crop) and the number of years of “good” acorn production immediately prior to harvest (1 year or 2 years). Within each subfigure (a)–(b), different letters represent significantly different means based on post-hoc Tukey HSD tests.

Output metric SEEDDENS was affected significantly by acorn production scenario (*F*_4, 435_ = 806, *p*<0.01) but not harvest type (*F*_2, 435_ = 2.82, *p* = 0.06) or interaction of scenario and harvest (*F*_8, 435_ = 1.08, *p* = 0.38). For SAPDENS, harvest and scenario main effects were significant (*F* = 49.6, 13.9 respectively; both *p<*0.01) but their interaction was not (*F* = 0.91, *p* = 0.51). Based on post-hoc multiple comparisons, both SEEDDENS and SAPDENS increased with number of good acorn production years prior to harvest and with the strength of a good year, and the effect was stronger for seedling density (Fig 4). The most extreme scenario (2 good acorn production years in the stand prior to harvest, each 2 standard deviations above the mean) significantly increased SAPDENS in clearcut harvests by an average of 26.5% and midstory removal by an average of 18.9% relative to the average scenario where acorn production varied randomly.

### Harvest and yearly variation in seed predation processes

**Fig 5:**
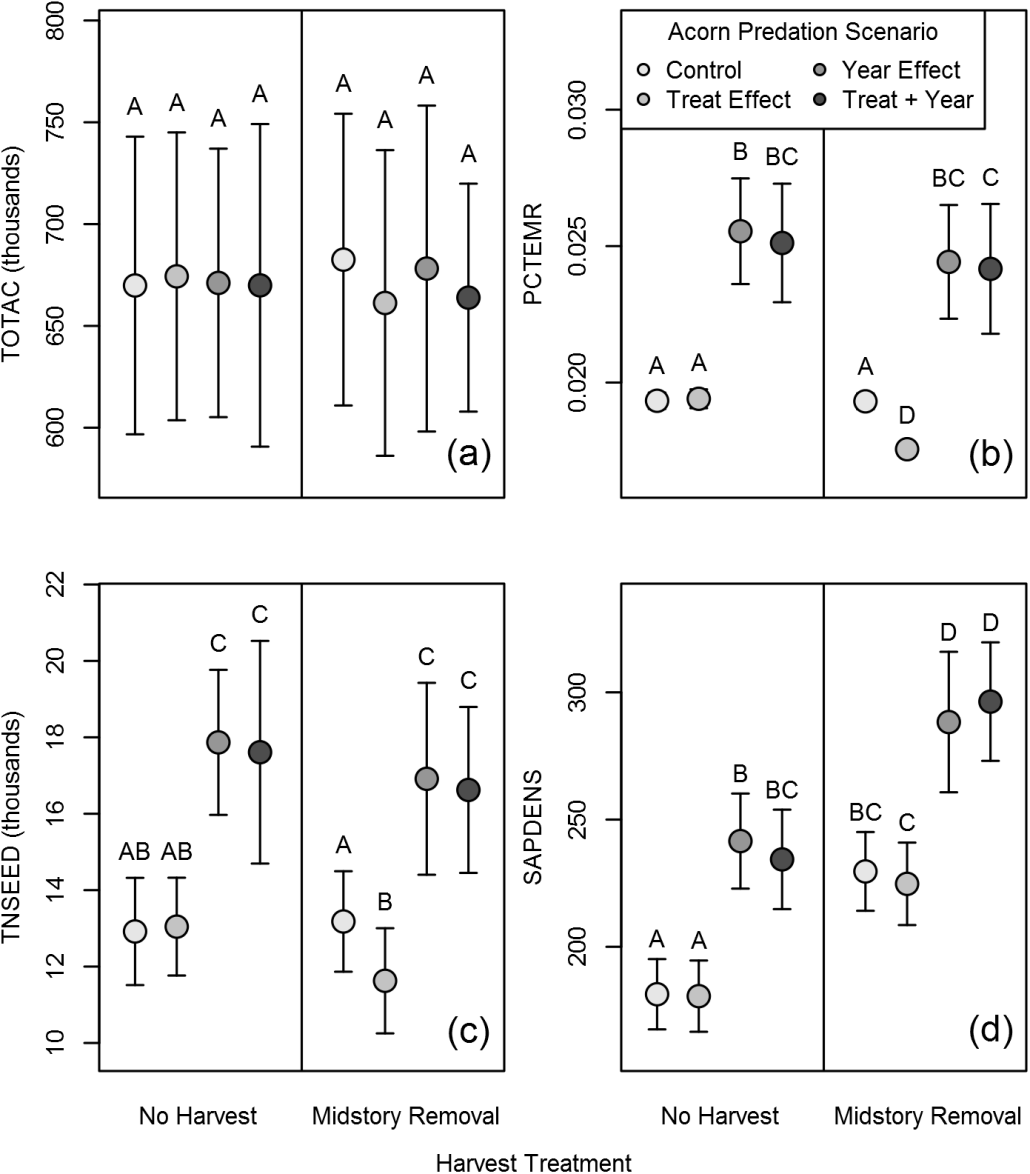
Effects of harvest and yearly variation on seed predation. Mean (± standard deviation) values for four oak regeneration metrics, under two harvesting treatments and four seed predation model scenarios: (1) a constant control; (2) harvest treatment effects on seed predation; (3) variable yearly effects on seed predation and (4) treatment and yearly effects together. Within each subfigure (a)–(d), different letters represent significantly different means based on post-hoc Tukey HSD tests.

Harvest treatment had significant effects on metrics PCTEMR, TNSEED, and SAPDENS but had no effect on TOTAC (Table 4). Across all scenarios, PCTEMR and TNSEED decreased by an average of 4% and 5% respectively in the midstory removal treatment. In contrast, SAPDENS increased by an average of 24% (Fig 5).

**Table 4:** Effects of harvest and yearly variation on seed predation.

		TOTAC		PCTEMR		TNSEED		SAPDENS	
Predictor	d.f.	*F*	*p*	*F*	*p*	*F*	*p*	*F*	*p*
Harvest	1, 232	0.00	0.99	24.0	<0.01	9.48	<0.01	415	<0.01
Scenario	3, 232	0.26	0.86	292	<0.01	111	<0.01	204	<0.01
Harvest × Scenario	3, 232	0.41	0.75	3.50	0.02	2.06	0.11	2.62	0.05

Two-way analysis of variance results for the effects of harvest treatment (clearcut or midstory removal) and seed predation scenario on four oak regeneration metrics. Results of post-hoc multiple comparisons of group (harvest treatment × scenario) means are found in Fig 5.

As with harvest treatment, scenario (treatment effects, yearly effects, or both included in acorn dispersal and survival regression models; Table 1) had a significant effect on PCTEMR, TNSEED, and SAPDENS but not on TOTAC (Table 4). Of the many possible post-hoc comparisons between scenarios, we were most interested in three: (1) inclusion of harvest treatment effects relative to the control in simulations that included a midstory removal; (2) inclusion of yearly-variable/mast-dependent effects relative to the control in both harvest treatments; and (3) inclusion of both harvest and yearly effects relative to the year effects alone in simulations that included a midstory removal. We found that following simulated midstory removal, inclusion of harvest treatment effects reduced PCTEMR and TNSEED by 9% and 12% respectively relative to the control, but there was no significant difference in SAPDENS (Fig 5). Across both harvest treatments, inclusion of year effects resulted in significant increases of 28–33% in PCTEMR, TNSEED, and SAPDENS relative to the control. When effects of treatment and year effects were combined in the regression models, results were similar to including year effects alone–increases of 27–31% in PCTEMR, TNSEED, and SAPDENS relative to the control (Fig 5). However, there was no significant difference in any of the metrics when comparing the combined treatment and year effects scenario with year effects only following simulated midstory removal (Fig 5).

### Interactions of drought and harvest

**Fig 6:**
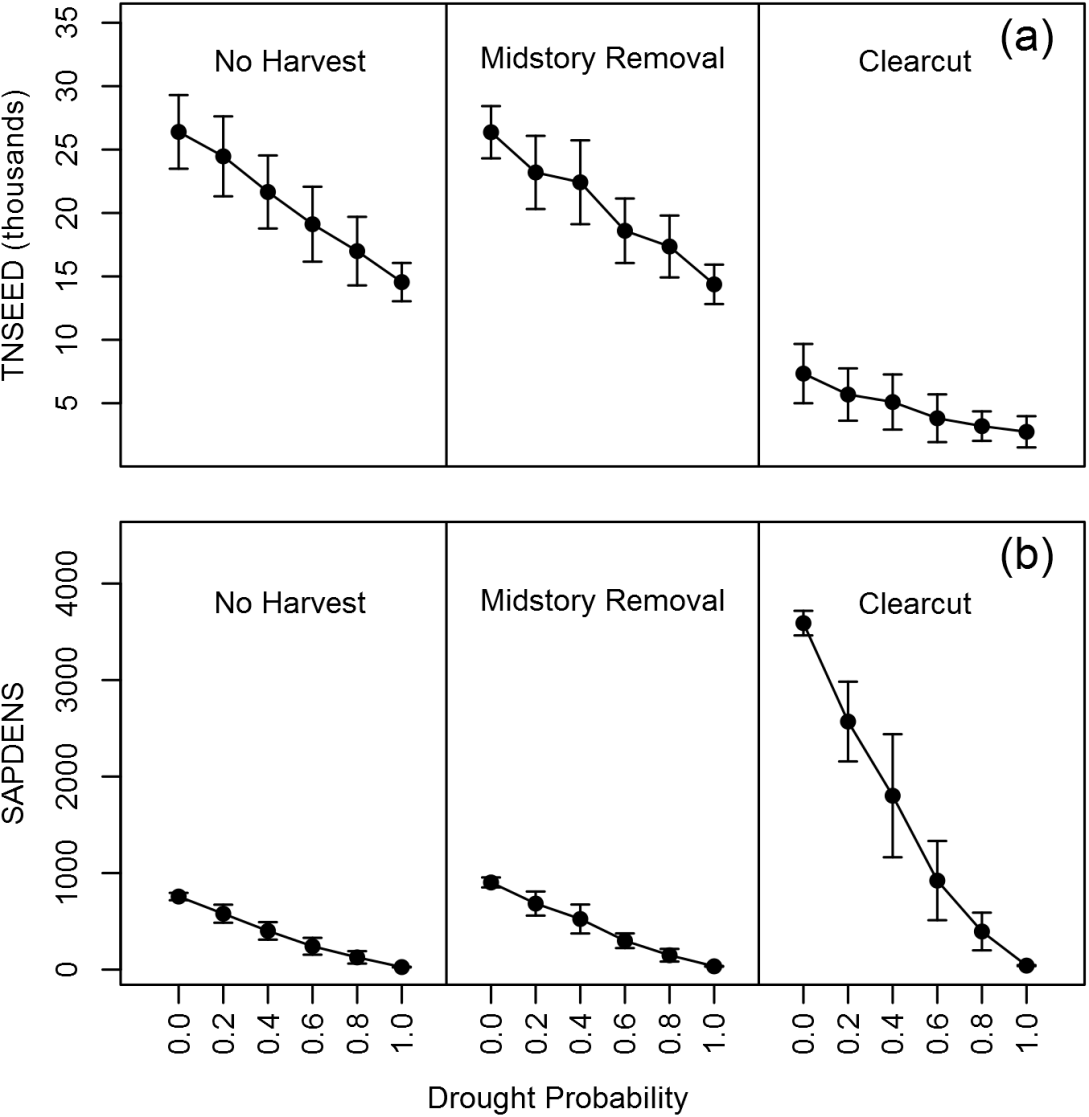
Effects of harvest and drought on oak regeneration. Mean (± standard deviation) values for TNSEED and SAPDENS under a range of yearly drought probabilities and three different timber harvest treatments.

There were significant main effects of both harvest treatment and yearly drought probability on TNSEED and the SAPDENS (Table 5, Fig 6). There were significantly fewer seedlings present post-harvest in the clearcut simulations relative to the other two treatments, and both the midstory removal and clearcut treatments had significantly higher SAPDENS (Table 5, Fig 6). Notably, the effect size of the clearcut harvest on SAPDENS was an order of magnitude higher than the effect of midstory removal (Table 5, Fig 6). As yearly drought probability increased, both metrics decreased significantly; each increase of 0.1 in the probability of drought decreased the expected TNSEED by 1203 per ha (a reduction of 4.5%) and the expected SAPDENS by 74 per ha (10.2%; Table 5). This negative relationship was not as strong for TNSEED in clearcuts (significant, positive interaction term between drought and clearcut harvest; Table 5) relative to the other two harvest types. However, increasing drought probability had a larger negative effect on SAPDENS in both clearcut and midstory removal simulations relative to no harvest (Table 5).

**Table 5:** Effects of harvest and drought on oak regeneration.

	TNSEED			SAPDENS		
Parameter	Estimate	*t-*value	*p*	Estimate	*t*-value	*p*
Intercept	26543.3	83.356	<0.01	724.5	22.082	<0.01
Midstory Removal	-348.5	-0.774	0.44	148.9	3.209	<0.01
Clearcut	-19635.4	-43.602	<0.01	2625.2	56.581	<0.01
Drought Probability	-12028.2	-22.873	<0.01	-738.6	-13.632	<0.01
Midstory Removal x Drought	410.6	0.552	0.58	-143.8	-1.877	0.06
Clearcut x Drought	7499.7	10.084	<0.01	-2854.3	-37.250	<0.01

Linear regression results for the effects of harvest treatment (none, clearcut, or midstory removal) and drought year probability on two oak regeneration metrics.

## Discussion

Forest succession depends on the presence and composition of advance regeneration in a forest stand, which in turn depends on biotic and abiotic filters along the pathway from seed to seedling [23, 69]. We synthesized field data into SOEL, a flexible individual-based model of the early oak life cycle (Fig 1) to quantify how key filters including seed predation, seed dispersal, and drought interact with silvicultural disturbance to influence the regeneration of oak. Three individual case studies demonstrate the ability of the model to connect intrinsic and environmental processes driving early oak life history with their ultimate consequences for oak regeneration.

### Interaction of harvest timing with acorn production

Oak regeneration metrics were sensitive to variability in the total number of acorns produced year-to-year (Table 3). High variability among years in seed production is characteristic of the oaks [1], and years of high acorn production are often followed by a high density of oak seedlings [16, 70]. Previous work on the efficacy of forest management practices for oak has demonstrated benefits from timing harvest to coincide with periods of high seedling density, thus maximizing the number of stems that have a chance to compete for light in openings[71]. Proper timing is difficult, given the challenge of predicting acorn production [72] and logistical constraints on when harvests are implemented. Our simulation results supported the conclusion that timing harvest to follow large acorn crops can measurably increase the density of oak regeneration in the short term, at least under some conditions (Fig 4). The impact of this acorn production “pulse” was strongly visible in the seedling density metric just before harvest for all scenarios; for the most extreme acorn production scenario seedling density was 126% higher than the control (Fig 4). The difference was not nearly as strong for sapling-sized oaks 7 years post-harvest, with the most extreme acorn production scenario yielding an average increase in sapling density of 22% across all harvest treatments (Fig 4). The difference is attributable to the increasing influence of other factors on oak seedlings over time (e.g. light availability, herbivory, and eventually density-dependent mortality among saplings).

Targeting forest management for oak to take advantage of multiple consecutive large acorn crops will not always be realistic. In the 9-year acorn production dataset that parameterized SOEL, mean white and black oak acorn production (per m^2^ canopy) exceeded one standard deviation above the mean just twice, and only approached two standard deviations above the mean once. Additionally, in the model, there were only two oak species, and they produced synchronous large acorn crops. In reality, different oak species (especially oaks in different sections) do not necessarily have matching year-to-year masting schedules [73–75]. Managers may not have the flexibility to wait multiple years for maximal acorn production to implement harvest, especially given the fairly modest increases in sapling density even in a perfect scenario (Fig 4). Avoiding bad acorn production years may be a more realistic criteria for timing harvest.

### Harvest and yearly variation in seed predation processes

At the acorn life stage, several life history parameters were affected by midstory removal (Table 1), though the effect (in terms of consequences for acorns) was not in a consistent direction. For example, midstory removal decreased probability of weevil infestation but increased probability of consumption by granivores [15, 20]. Given these variable effects do these harvest effects, pooled across the entire early oak life history, have a measurable effect on metrics of oak regeneration?

The answer depends on the focal oak life stage. In the absence of yearly variability, the cumulative impact of the midstory removal, across all acorn parameters, was a small but significant reduction (9–12%) in the probability a given acorn established into a seedling and the total number of new seedlings that accumulated across the 7-year post-harvest period (Fig 5). The difference became negligible at the sapling life stage (Fig 5). Thus, simulation results suggest that acorn predator-midstory removal interactions have a measurable effect on the oak regeneration process initially, but these effects decline over time and thus likely do not drive the dominance of oak in the future canopy. One potential explanation is that increased sapling growth and survival in the higher-light environment of the midstory removal offsets any negative effects of the harvest at the acorn life stage. The overall weak impact of partial midstory removal is not surprising given the modest level of disturbance to the forest stand [43]. We only examined the effects of harvest on predation through the initial phase of a three-phase shelterwood. Additional work is needed to determine if the more intensive disturbance in the later phases of the shelterwood harvest (i.e., greater amounts of basal area removal) have stronger effects on acorn-level life stage parameters of oak.

The impact of including year-to-year variation in acorn-level parameters was an increase in the mean value for all three metrics of oak regeneration by roughly a third, with a commensurate increase in variability of the regeneration response. The magnitude of these changes, relative to scenarios in which year-to-year variation was absent, were such that they masked the much smaller effects of harvest on oak regeneration (Fig 5). A primary driver of year-to-year variability in parameter values was the mean acorn input into the model system, which had an overall negative correlation with several metrics of acorn predation (Table 1). While predator populations were not modeled explicitly, this negative relationship implicitly includes the impact of predator satiation [2, 15–16]. In years with large acorn crops, overall seed predation was reduced and more acorns escaped to germinate into seedlings (Fig 5).

### Interactions of drought and harvest

We previously demonstrated differences in seedling growth and survival in drought years, especially in recent clearcut harvests [39]. Specifically, drought changed the predictive relationship between shade and seedling survival and growth. In drought years, seedlings in high-light environments had lower survival and growth, presumably because they were under greater water stress [56, 76]. Given the decrease in survival and growth in drought years for a given level of light, the ultimate consequence of increasing yearly drought probability was a large reduction in both the seedling and sapling regeneration metrics to the point where essentially no seedlings reached the sapling size category (> 1.4 m height) when there was a 100% probability of drought (Fig 6). The impact of drought was greater for black oak relative to white oak (which reflects the field data on which the model is based); when drought year probability was 0, 30% of the final density of oak saplings were black oaks, whereas when drought probability was 0.8, only 24% were black oak. Thus when drought is frequent, high-light environments represent a tough trade-off for oak seedlings between growth and survival, and there are species-specific differences in overall response even with the genus *Quercus*.

While there are clearly negative consequences of drought for oak regeneration, the impact of drought is likely to be even greater for competing tree species that do not have oak’s adaptations to xeric environments [9]. Drought could therefore facilitate competitive success of oak, particularly in harvest openings where competition from pioneer tree species is fierce [77]. In the drought case study, drought impacted only oak seedlings and saplings and not the competing maple and tulip poplar saplings. A logical extension of this simulation would be to use field data to parameterize the impacts of drought on competing saplings as well, thus yielding a more realistic composition of regeneration in different drought scenarios.

More frequent droughts (along with higher temperatures) are predicted for eastern U.S. forests due to climate change [58]. The impacts of climate change on forests have been simulated at large spatial scales (e.g. [78]), but there is less focus on finer-scale inference. This case study, examining the impact of drought, represents a first step in simulating the impacts of climate change on oak regeneration. Tying the frequency of drought years in the simulation to predictions from climate models would give managers useful information on expected oak regeneration outcomes from different harvesting regimes in the future. Additional field data that allowed for incorporation of continuous climate data in predictive models of oak seedling growth and survival (in contrast to the binary drought/non-drought predictor used in this case study) would allow for more accurate predictions.

### Conclusion

Individual-based modeling provides a useful, spatially explicit framework for integrating multiple datasets across life stages to examine questions about the tree regeneration process. Using SOEL, we quantified the importance of multiple parameters impacting early oak life history (Fig 1), and confirmed via three case studies that both intrinsic and extrinsic processes influenced early oak life history and thus the oak regeneration process. At the acorn life stage, acorn production was positively related to the system both directly and indirectly by altering variability in the probability of acorn infestation and predation (Fig 5). Additionally, the interaction of acorn production with harvest (i.e., timing harvest to coincide with large acorn crops) continued to have measurable effects under the most extreme scenarios on sapling density multiple years later (Fig 4). At the seedling life stage, the interactive effects of light, drought, and harvest drove growth and survival.

The case studies presented here represent only a few straightforward examples of what could be accomplished within the framework of SOEL. Further studies could put more focus on different early life history parameters (Fig 1), or connect the parameter values to different predictor variables based on field data. For example, our case studies did not focus on caching probability despite the fact that variability in caching had a strong effect on oak regeneration parameters (Table 3). With appropriate field data, the model could be extended to assign utility to individual acorns based on seed characteristics (species, size, nutrient composition) [79], incorporate effects of relative frequency of different seeds on hoarding [80], and model caching probability as a function of these attributes Likewise, the three cases studies had a minimal emphasis on the impact of browse damage on seedling growth and survival, despite the demonstrated ability of herbivores (particularly white-tailed deer) to greatly impact oak regeneration [28, 32, 81]. Deer densities, and overall rates of herbivory, were low across our study area [31, 82]. In areas where deer are more numerous and exert more control over the regeneration process, SOEL could be used to examine effects of browse damage that vary spatially across harvest opening edges [56]. Finally, we recognize a need to incorporate additional data at the seedling and sapling stages of the model, particularly effects of intra- and interspecific competition between seedlings and stump sprout growth and survival.

## Supporting information

S1 Appendix Detailed description of SOEL model structure and parameter estimates. (PDF)

S1 Code NetLogo and R source code for SOEL and JABOWA. (ZIP)

S2 Code R code to fit regression models for individual oak life history parameters. (ZIP)
