# Supplementary material for: Simulation of oak early life history and interactions with disturbance via an individual-based model, SOEL
Source: PLoS One. 2017 Jun 20;12(6):e0179643. doi: 10.1371/journal.pone.0179643 (PMC5478140; doi:10.1371/journal.pone.0179643)
Supplement: S1 Appendix — (PDF) [file pone.0179643.s001.pdf]

S1 **APPENDIX**: SOEL MODEL DESCRIPTION

Kellner KF[1], Swihart RK[1]. 2017. Simulation of oak early life history and interactions with disturbance via an individual-based model, SOEL. PLOS ONE.

CONTENTS

1 Introduction 1
2 Selection of a Modeling Framework 6
3 Model Overview 6
3.1 Purpose . . . 8
3.2 Entities, State Variables, and Scales . . . 8
3.3 Process Overview and Scheduling . . . 9
3.4 Design Concepts . . . 9
3.5 Initialization . . . 10
3.6 Input . . . 11
4 Early Life History Submodel 11
4.1 Acorn Production . . . 11
4.2 Acorn Predation, Dispersal, and Germination . . . 12
4.3 Oak Seedling Growth and Survival . . . 12
5 Contextual Forest Submodel 13
5.1 Calculate Environmental Conditions . . . 13
5.2 Growth . . . 16
5.3 Survival . . . 20
5.4 Reproduction . . . 20
5.5 Timber Harvest . . . 21
6 Conclusion 23
7 References 23

1 INTRODUCTION

Understanding the development and function of forests is a significant research challenge. Processes that drive tree reproduction (e.g. seed predation, dispersal, seedling herbivory,

[1] *Department of Forestry and Natural Resources, Purdue University, West Lafayette, IN*

competition) occur on a short time scale relative to the potential lifespan of a tree (hundreds to thousands of years). Thus, the ultimate consequences of small perturbations to a forest ecosystem may take centuries to manifest. Empirical forest research typically has been limited to much shorter time intervals, which makes it difficult to ascertain what factors caused a forest to reach its current state and (perhaps more importantly) what the forest will look like in the future. Some forest research projects have collected or are working to collect long-term datasets [e.g. 1, 2, 3, 4, 5], but these projects are arguably the exception and not the rule. Historical patterns can be reconstructed using natural records like tree rings [6], fire scar chronologies [7, 8], pollen records [9], or even woodrat middens [10]. These approaches can help us understand trends in tree growth, climate, and vegetation composition over time but are less useful for understanding interactions between trees and other organisms or predicting the effects of new management strategies. To address the latter issues and many others, forest researchers have often turned to simulation modeling [11, 12, 13]. While modeling allows for great freedom in answering research questions, the complexity of the questions and the time interval in which they are addressed are limited by computational power and by the modeling framework (i.e., structure and parameterization). Thus, a wide variety of modeling frameworks have been developed, each with strengths and weaknesses, to address different types of forest research questions.

These forest modeling frameworks can be organized conceptually by the degree to which a model incorporates details about individual organisms. At one end of this conceptual scale are forest ecosystem (or forest landscape) models [12]. Modeling detailed dynamics of individual trees (and/or individual tree species) is not a goal of these models; instead they focus on overall stand density, composition, and biomass. This approach sacrifices individual detail for tractability in estimating large-scale, long-term patterns at a landscape scale (i.e., effects of climate change or carbon fixation). Probably the best example is the LANDIS II model developed by Eric Gustafson, R. J. Scheller, and colleagues [14].

Matrix projection models track counts of individual organisms in discrete age, size, or life stage classes. Within a given class, all individuals are considered identical. Matrices of transition probabilities (vital rates) are parameterized to describe how individuals move between the categories. Useful information about population dynamics is straightforward to obtain from matrix models; for example, the populations asymptotic growth rate λ and the stable age/size distribution can be calculated as the dominant eigenvalue and right dominant eigenvector, re-

spectively, of the transition matrix [15]. These parameters are useful for assessing the trajectory of the population and how it is affected by different vital rates. Thus, they have been applied to a wide variety of plants and animals since they were introduced [16] including trees [17].

For long-lived, relatively slow-growing organisms like trees, matrix projection models do have some disadvantages. First, trees in matrix models are usually classified based on size (e.g., height or diameter at breast height), even though size actually varies continuously [18]. Thus, heterogeneous individuals in a given size class will be treated as identical, which introduces error. Increasing the number of discrete classes can reduce this error, at the cost of reduced sample size and therefore reduced precision in transition probability estimation [19]. Changing the number of discrete classes in the model can also influence elasticity and sensitivity measurements [20]. Second, matrix models have only limited ability to incorporate variability in growth rates among individuals [18]. For long-lived species especially, individual variability in growth may strongly influence population growth rate [21].

Integral projection models (IPMs) are a logical extension to matrix models that address these disadvantages. The key difference between IPMs and matrix models is that while vital rates in matrix models are class-specific, in IPMs vital rates can be functions of the underlying continuous trait (e.g., size) [22]. This characteristic allows IPMs to incorporate individual variability in vital rates and relax the requirements for discrete classes [18]. IPMs address these limitations of matrix models while still yielding similar information about population dynamics including population growth rate and stable size distribution [19]. While IPMs are flexible overall, some ecological processes are currently more challenging to incorporate into the IPM framework, including spatially explicit variability and dispersal [23, 24, *but see* 25].

Individual-based models (IBMs; also called agent-based models) use a simulation approach to examine how an overall system (e.g. a population or community of organisms) affects, and is affected by, its autonomous component agents [26]. In ecology, they share a general structure with analytical approaches like matrix and integral projection models in that they are driven by an underlying set of ecological processes (e.g. survival and growth). Individual-based models allow for straightforward inclusion of spatially explicit processes, interaction between individuals, and adaptive behavior of individual agents, which are challenging to implement in analytical modeling approaches [26]. However, because they are based on simulation they give up some of the analytic results that can be obtained from matrix and integral projection

modeling [25]. Additionally, they typically require a large number of parameters and substantial computing power [26].

IBMs have a long history in forest modeling and have become increasingly popular in ecology in recent years owing to new software tailored to IBM analysis and increases in computing power [26]. Forest IBMs can take many forms but typically consist of a schedule of submodels or rules that govern the vital rates, movement, and interaction of individuals with each other and the environment over a series of time steps [13, 26]. Among the first and most influential forest IBMs was JABOWA, which inspired a class of IBMs called gap models [27]. The basic structure of JABOWA consists of a number of independent 10 $\times$ 10 m cells, each containing a set of trees that regenerate and grow according to species-specific rules and compete for light with other trees in the same cell [27]. JABOWA was widely implemented, in part because of the ease with which it is parameterized (only 5-10 parameters per tree species are required), and also because the independent cells can be simulated sequentially to cut down on computing power required [13].

Of course, the simplicity of the model detracts from realism; JABOWA makes many assumptions about the shape of tree crowns, trees in adjacent cells do not interact in any way, and ecological processes connecting adult and sapling trees (seed production, dispersal, etc.) are ignored. Other gap models built upon the foundation of JABOWA while adding additional realism, including FORET [28] and ZELIG [29]. Among other additions, ZELIG added more detail to the simulation of tree crown shapes and allowed for large trees to shade adjacent cells [30]. ZELIG has been further modified to examine the effects of herbivory on tree regeneration [31, 32]. Even more complex forest IBMs like SORTIE have taken advantage of modern increases in computing power to offer a highly realistic representation of forest structure and plug-ins to simulate numerous ecological processes that drive forest dynamics, including seed production, dispersal, and herbivory [33]. Of course, in addition to their computational requirements, these more detailed modeling frameworks require many more parameters to be estimated (either from the literature or via experiments) than the original types of gap models and must be carefully calibrated to a specific region and forest type; thus, they may not be the most logical choice for simpler studies of forest dynamics.

Forest simulation models have been employed to answer a wide variety of research questions across many study systems, regions, and forest types [34, 34], but fine-scale ecological processes driving dynamics of early-tree life stages (e.g. seed predation, seed dispersal, and

herbivory) have not been a major modeling focus [*but see* e.g. 31, 32]. One system that would benefit from a better understanding of how these processes ultimately influence regeneration and forest succession is the central hardwood forest region of the eastern United States [35]. Oak (*Quercus*) is a dominant canopy species in this forest region providing key value to wildlife as a food resource [36, 37] and to humans as a timber species [38]. While dominant in the canopy, oak is typically absent from the forest midstory and understory in the central hardwoods; over time this is expected to result in oak being replaced in the forest canopy by more shade-tolerant tree species (oak regeneration failure; [39, 40]).

A primary culprit in oak regeneration failure is shifts in disturbance regimes, namely suppression of wildfires that favored regeneration of fire-adapted oak [41]. Timber harvesting is one way of reintroducing disturbance to promote oak regeneration [42], and a number of different silvicultural techniques have been developed to do so, with mixed success [43, 44]. However, oak regeneration is also challenged by a multitude of other factors, including a large suite of seed predators and herbivores that rely on oak acorns and oak seedlings for food [36, 37, 45]. Acorn-seed predator and oak seedling-herbivore interactions may themselves be affected by timber harvesting disturbance [e.g. 46, 47, 48, 49, 50]. Understanding and predicting the outcome of these trophic interactions is challenging, but would be valuable for designing silvicultural prescriptions that promote oak regeneration.

The ecological processes occurring in the early life stages of oak (i.e., acorn to seedling) occur over a short interval of time (a few years) relative to the oak life expectancy (hundreds of years). Observed and experimental data related to the early life history of oak is often similarly collected over short periods of time. This temporal and logistical limitation of oak studies makes it difficult to project the ultimate consequences of factors affecting early oak life stages on the final population or forest stand. We developed a model to bridge that gap; i.e., a simulation-based approach to determine how changes in parameters of oak early life stages ultimately affect the persistence of oak as a canopy dominant species in managed forest stands hundred(s) of years later.

2 SELECTION OF A MODELING FRAMEWORK

The model structure was designed with several important considerations in mind. First, empirical data for parameterizing early oak life history processes were collected at the scale of individual acorns and seedlings [50, 51, 52], meaning the forest ecosystem modeling approach (e.g. LANDIS), which occurs at the stand scale and does not track individual trees, was not appropriate. Second, early oak life history involves several spatially-explicit processes including acorn dispersal and interaction between oak seedlings and nearby competitors (primarily competition for light). Given these considerations, we judged an individual-based simulation model to be the most straightforward approach to incorporating field data into a spatially-explicit modeling framework.

We therefore developed a spatially-explicit, individually-based model called SOEL: **S**imulation of **O**ak **E**arly **L**ife history. SOEL is composed of two primary submodels: the early life history submodel, and the contextual forest submodel simulating the forest in which the early life history submodel occurs (Figure 1). The early oak life history submodel tracks oaks from acorn to sapling and is governed by a set of key parameters parameterized using field data [50, 51, 52]. The contextual forest submodel simulates growth and mortality of sapling-size and larger trees. We did not have field data to parameterize the contextual forest submodel; thus, we adopted an existing modeling framework to do so, JABOWA [27]. JABOWA was selected due to its widespread use yet relatively simple parameterization; it was modified in several ways to match the spatially-explicit structure of the early oak life history submodel. An overview of the model is given in section 3, followed by a detailed description of the early life history submodel in section 4 and the contextual forest submodel in section 5.

3 MODEL OVERVIEW

SOEL is summarized below using the ODD (Overview, Design concepts, Details) protocol [53] and was implemented in the NetLogo 5.2.0 programming language [54]. Model code for SOEL is provided as supplementary information (S2 SOEL Code).

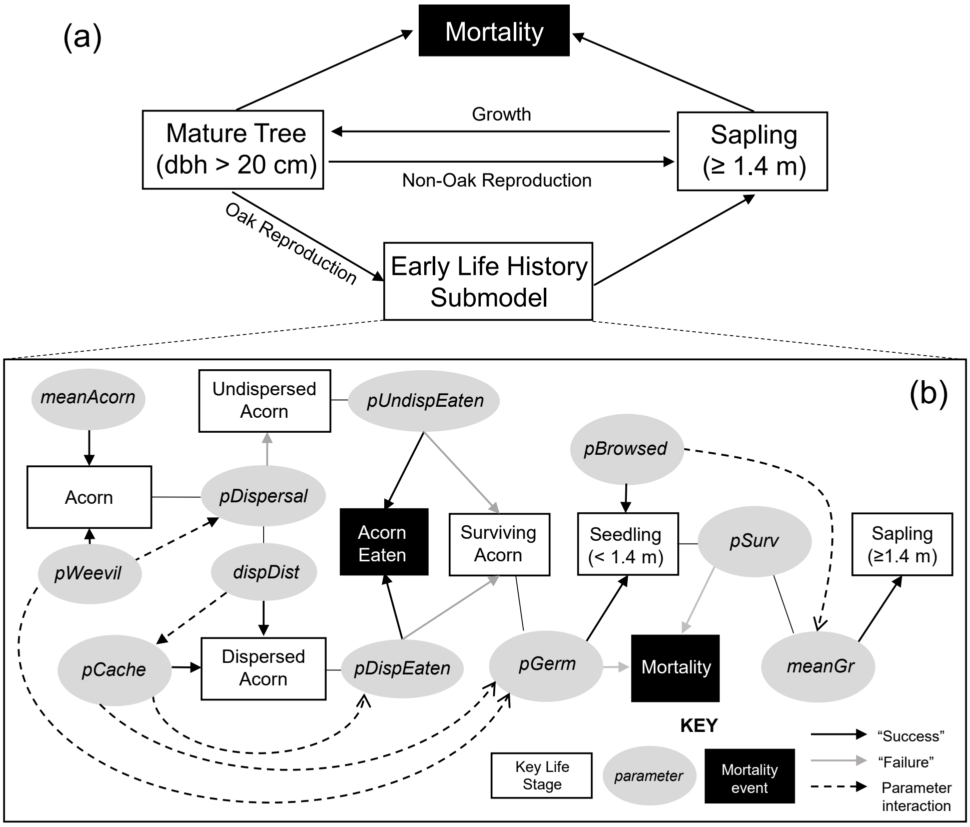

Figure 1: Overview of SOEL structure including (a) the contextual forest submodel derived from JABOWA and (b) the early life history submodel parameterized with field data. Key parameters in (b) are in gray ovals. For probability parameters in (b), black arrows indicate the result of a "success" and gray arrows the result of a "failure".

3.1 Purpose

The purpose of SOEL is to determine how processes involved in the early life history of oak (e.g., seed production, predation, dispersal, competition and herbivory) interact with timber harvest to drive oak regeneration, and ultimately forest succession, following harvest in a multi-species stand.

3.2 Entities, State Variables, and Scales

There are three types of entities included in SOEL: patches, trees, and seeds (specifically, acorns). Patches are 1 $\times$ 1 m in size and have one primary state variable: the amount of canopy cover in that patch. Canopy cover values range from 0 to 1, where 1 represents complete shade from tree canopies (no light passing through). These cover values are calculated based on the height and canopy radius of the trees in and around each patch and are used to calculate the light available to each tree. The simulated area consists of a core square area of patches surrounded by a buffer, both of which can be variable in size. The default is a 100 $\times$ 100 core grid of patches (1 ha) surrounded by a 20 patch (20 m) buffer on all sides for a total simulated area of 1.96 ha.

Trees in the model are treated differently based on species, of which there are four: white oak, black oak, tulip poplar, and sugar maple. Tulip poplar was included in the model to represent oak competitors in recently harvested areas [55], while maple was included to represent oak competitors under mature forest canopies [40]. Each tree species has its own set of parameters governing growth, survival, and reproduction under different environmental conditions. Oaks, the main focus of the model, are simulated in more detail than the other species. This difference manifests in two ways. First, oaks have three life stage categories: seeds (acorns), seedlings (<1.4 m tall), and sapling/adult trees ($\geq$ 1.4 m tall). For sugar maple and tulip poplar, only sapling/adult trees are simulated. All life-stage categories have a state variable associated with spatial location within the simulated stand. Oak acorns have one additional primary binary state variable to indicate weevil infestation status. Oak seedlings are characterized by a single additional state variable, height. Sapling/adult trees have several additional state variables: (1) height; (2) diameter at breast height, dbh; (3) canopy radius, since canopies are assumed to be

circular and flat; and (4) age. Oaks additionally have (5) fecundity in terms of average number of acorns produced per unit canopy area.

3.3 Process Overview and Scheduling

Each model time step represents one year. Broadly, four processes run in each time step in the following order: (1) tree growth (2) tree survival (3) tree reproduction (4) timber harvest, if applicable (Figure 1). The tree growth and survival process for sapling/adult trees (all species) is separate from the process for seedlings (oaks only; Figure 1); both depend on site characteristics, light availability, and species-specific parameters. Reproduction for oaks involves several sub-processes including acorn production, predation, dispersal, and germination (Figure 1). For the other tree species, reproduction is simpler and uses the JABOWA approach - saplings are spawned on patches directly, skipping the earlier life stages. When a timber harvest is conducted, trees are removed (equivalent to mortality) from the simulation based on a set of rules specific to the timber harvesting type; the timber harvesting options are clearcut (all trees removed every 100 years), shelterwood (all trees removed in three phases every 100 years), and single-tree selection (a subset of individual merchantable trees removed each year). The individual submodels are described in greater detail in sections 4-5.

3.4 Design Concepts

In SOEL, light availability is the most important factor driving tree survival and growth. Trees implicitly sense light availability in the environment based on their height, and their growth and survival is scaled accordingly. The height and spatial configuration of trees leads to a key interaction influencing light availability; trees reduce the light available to surrounding shorter competitors (i.e., shade), and in turn are shaded by taller competitors. When stem density is high, individual trees will have little light available and so growth will slow and density-dependent mortality will be high. However, there are species-specific differences in the ability to tolerate shade so some tree species (e.g., tulip poplar) are more negatively affected by shade than others (e.g. sugar maple). Thus, via changes in survival and growth, abundance and distribu-

tion of each tree species is an emergent property of its ability to grow and survive under light conditions which vary across the landscape, with time, and with timber harvesting treatment.

3.5 Initialization

The IBM is initialized by creating a mature (80-100 year old) forest stand. The basal area, dbh distribution, and species distribution of the tree species are based on pre-harvest forest survey data from the Hardwood Ecosystem Experiment (HEE), in the forest system on which the IBM is based [56]. The HEE tree distribution data (which included many more tree species than we are including in SOEL) was divided into three categories: oaks, shade-tolerant species, and shade-intolerant species (Table 1). An equal number of black and white oak trees of appropriate size were initialized to match the HEE data for oaks. Tulip poplars and sugar maples of appropriate size were initialized to match the basal area and size distribution of the pooled shade-intolerant and shade-tolerant species groups, respectively. Overstory trees of all species are assigned locations within the simulation in a random order; the assigned location is also random, subject to the constraint that the location is >7 m from any other mature trees (to avoid unrealistic clusters of overstory trees). Midstory and understory oaks and tulip poplars are assigned random locations subject to the constraint that the location has at least 60% full sunlight. Maple midstory and understory trees are assigned completely random locations throughout the simulation. Before any experiments are conducted on the simulated forest, a 30 time-step (30-year) burn-in is conducted to minimize the effects of the initial conditions.

Table 1: Values used to initialize the forest stand in each model run. The initial forest included trees in two size categories (midstory and overstory) for each of the three tree categories in the model: oak (white oak *Quercus alba* and black oak *Q. velutina*), shade tolerant (sugar maple *Acer saccharum*) and shade intolerant (tulip poplar *Liriodendron tulipifera*). Each size $\times$ species category was defined by an initial density (per hectare) and diameter at breast height (dbh) distribution. Values are based on forest structure data collected pre-harvest from the Hardwood Ecosystem Experiment [56].

			dbh	
**Species**	**Size Category**	**Trees ha^{-1}**	**Mean**	**SD**
Oaks (both)	Midstory	95	14.9	5
	Overstory	89	45.75	5
Sugar maple	Midstory	499	10.3	5
	Overstory	11	40.8	5
Tulip poplar	Midstory	163	14.9	5
	Overstory	9	45.07	5

3.6 Input

All parameter values and initial conditions are controlled within the NetLogo modeling interface; no external data is read into the model. There are several primary variables set within NetLogo before each model run. First, the size of the simulated forest stand and the buffer around the stand are specified. Second, site conditions are specified (mean temperature, moisture, and soil fertility). Finally, the timber harvest treatment (clearcut, shelterwood, single-tree selection, or no harvest) is selected.

4 EARLY LIFE HISTORY SUBMODEL

4.1 Acorn Production

Oaks are a masting species, characterized by a boom-and-bust cycle of acorn production [57]. To simulate that process, each time step in the model is assigned a different value for mean acorn production per m^2 of canopy area, parameter *meanAcorn* (Figure 1). The value of *meanAcorn* is based on field data [50] and can be fixed or follow a set pattern (for example, a sequence of average, good, and poor mast crop years). Table 2 gives a set of example values for *meanAcorn*. Within each year, every oak is assigned a random value for acorns produced per m^2 of canopy area from an exponential distribution with mean meanAcorn. The tree then drops a number of acorns underneath its canopy equal to this random value multiplied by its canopy area. In some oak species (including white oak), acorn production declines after a peak size is reached [58]. This decline was not incorporated into the model because we did not have field data on acorn production from very large, very old oaks and we were more interested in production by trees around the age typically observed in a managed forest (80-120 years) when harvesting takes place.

Table 2: Yearly mean acorn production parameter *meanAcorn* (per m^2 canopy area) by black (*Quercus velutina*) and white (*Q. alba*) oaks under three masting scenarios: low mast year, average mast year, and high mast year. Mast production by individual trees each year is randomly selected from an exponential distribution with mean equal to *meanAcorn*. Total acorn production means by average-sized trees (canopy radius 5 m) are presented for comparison.

	**Low Mast Year**		**Average Mast Year**		**High Mast Year**	
**Species**	***meanAcorn***	**Total**	***meanAcorn***	**Total**	***meanAcorn***	**Total**
Black Oak	3.75	294	11.07	869	16.58	1303
White Oak	8.58	673	11.74	922	22.34	1754

4.2 Acorn Predation, Dispersal, and Germination

After acorns are produced, they can be predated by acorn weevils [59] and small mammals [46, 50], and may also be dispersed by small mammals [60, 61]. In the basic version of SOEL described here, these probabilities of predation and dispersal are fixed, based on empirical data from the Hardwood Ecosystem Experiment [50, 52]; however they can be varied as functions of other covariates depending on the research goal. Infestation occurs first with probability *pWeevil*; weevil infested acorns are not dispersed but may be consumed by predators [62]. Uninfested acorns may be either left where they fell, or dispersed with probability *pDispersal*. The dispersal kernel is assumed to be isotropic; distance is a random value from a Weibull distribution with scale parameter *dispDist* and shape parameter fixed at 1.4. Dispersed acorns are cached with probability *pCache*. Acorns that were not dispersed are consumed by granivores with probability *pUndispEaten* and acorns that were dispersed are consumed with probability *pDispEaten*. Dispersal and caching parameters are based on HEE data [52].

All acorns that escape predation (either dispersed or not) have a probability *pGerm* of germinating into a seedling. Germination probability was obtained from the literature and was higher for acorns that have been cached and lower for acorns that are infested by weevils [63, 64]. Acorns that do not germinate in the year in which they are produced are removed from the simulation (i.e., there is no year-to-year seed bank for acorns).

4.3 Oak Seedling Growth and Survival

The expected growth of a given seedling *meanGr* depends on oak species, available light (see section 5.1), and the presence of browse damage in a given year which occurs with fixed probability *pBrowse* (Figure 1; [65]). A predictive growth model was fit using empirical data

[66] as a function of oak species *sp* (binary variable with white oak = 1), percentage of shade cover *sh* (see section 5.1 for calculation), presence/absence of browse damage *br*, a random seedling effect $rs \sim N(0, 0.25)$ and residual error $\epsilon \sim N(0, 1.19)$. We transformed the empirical growth data using the neg-log transformation [67], thus yearly seedling growth was calculated as:

$$\text{Growth} = \begin{cases} 1 - \exp\left(-1 \times \mu\right) & \text{if } \mu \leq 1 \\ \exp\left(\mu - 1\right) & \text{if } \mu > 0 \end{cases} \tag{1}$$

where

$$\mu = 1.24 - 0.48 \times sh + 0.12 \times sp - 0.94 \times br + rs + \epsilon \tag{2}$$

The probability of oak seedling survival *meanSurv* was also based on empirical data [66]. Survival probability ψ for a given year is a function of oak species *sp* (binary variable with white oak = 1), seedling age, and shade *sh*:

$$\psi = \frac{1}{1 + e^{-\mu}} \tag{3}$$

where

$$\mu = -0.60 + 0.10 \times sp + 0.37 \times sh + 0.58 \times age \tag{4}$$

5 CONTEXTUAL FOREST SUBMODEL

5.1 Calculate Environmental Conditions

Growth, survival, and regeneration of mature trees in the JABOWA-derived contextual forest submodel depend on two key environmental conditions: site quality and light availability. During model initialization, a species-specific metric of site quality *qE* is calculated according to [27]. Site quality is based on four environmental characteristics, assumed constant across space and time in the simulation: (1) site climate (temperature), measured as the number of degree-days at the site; (2) soil fertility, measured as kg/ha nitrogen; (3) soil moisture (or wilt potential), measured based on a comparison between potential and actual evapotranspiration; and (4) soil water saturation measured as depth to the water table (m). The effect of each en-

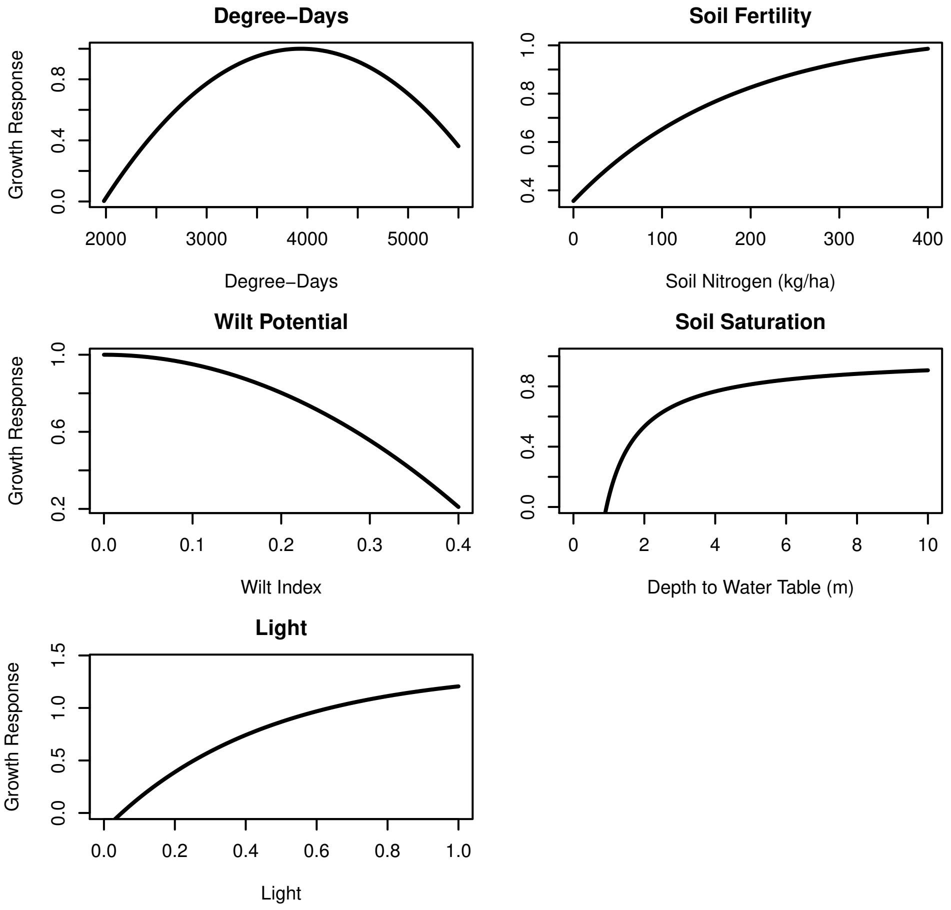

Figure 2: White oak (*Quercus alba*) growth response function curves for five environmental characteristics (1) degree-days at the site; (2) soil fertility, measured as kg/ha nitrogen; (3) wilt potential, measured based on a comparison between potential and actual evapotranspiration; (4) soil water saturation measured as depth to the water table (m); and (5) proportion of full light available. The product of the first four response functions is the overall site quality index *fQ* for white oak.

vironmental variable is obtained from response functions bounded on [0,1] where 1 represents ideal conditions for tree growth and survival (Figure 2). Each response function is parameterized with species-specific values (Table 3), so ideal conditions may differ between species. The site quality *fQ* for each species is calculated as the product of the values from the four response functions and is therefore also bounded on [0,1] with 1 representing ideal site quality for the species.

Available light (ranges from 0 to 1) is calculated for each individual seedling, sapling, and adult tree. The baseline light availability above the canopy (ϕ) is set to 1 in every patch at the beginning of each time step. Simulated trees have canopies in which all of the leaf area is compressed into a flat disk of known radius located at the top of the tree (similar to [27]). Beginning with the tallest tree in the simulation, available light is calculated as the mean light

Table 3: Parameter estimates for the contextual forest submodel of SOEL for the four tree species included: black and white oak (*Quercus velutina* and *Q. alba*), sugar maple (*Acer saccharum*), and tulip poplar (*Liriodendron tulipifera*). Parameter estimates were obtained from [27, 32, 50].

Parameter	Description	Black Oak	White Oak	Sugar Maple	Tulip Poplar
DBH_{max}	Max possible tree DBH (cm)	100	100	100	100
H_{max}	Max possible tree height (cm)	3800	3800	3350	4000
Age_{max}	Max possible tree age (years)	300	400	400	300
C	DBH-leaf area relationship	1.75	1.75	1.57	1.75
G	Controls growth rate	122	104	119	140
L	Tolerance for low-light conditions	Intermediate	Intermediate	High	Low
$DEGD_{min}$	Minimum tolerable degree-days	1977	2068	2000	2171
$DEGD_{max}$	Maximum tolerable degree-days	5984	5421	6300	6363
N	Tolerance for low soil nitrogen (fertility)	Intermediate	Intermediate	Intermediate	Intermediate
DT_{min}	Min tolerable depth to water table (m)	0.933	0.933	0.567	0.544
WILT	Wilting index	0.45	0.45	0.35	0.245
S	Max saplings/year entering per 100 m^2	N/A	N/A	3	10

available in patches that overlap the trees canopy; by definition, for the tallest tree, this will be 1. The shade cast by the first tree is then determined. The tree canopy shades individual patches underneath it according to the Beer-Lambert law [68]. The amount of light that passes through the canopy (*AL*) is a function of the trees leaf area index (*LAI*, itself a function of dbh) and a light extinction coefficient (k = 1/4000; [27]):

$$AL = \phi \times e^{-k \times LAI} \tag{5}$$

Shade is then calculated as 1 - *AL*.

If the suns position were fixed and directly overhead, the tree canopy would shade a circular region of patches directly underneath it at all times; this was the approach of JABOWA-3 (although the shaded area in JABOWA was the entire square cell instead of a circular region based on tree size) [27]. In reality, as the time of day, date, and latitude change, the angle of the canopy relative to the sun changes and therefore the exact area shaded relative to the tree shifts. This distinction is particularly important when simulating timber harvests (or other disturbance events), since a perfectly vertical shade profile underestimates the shade cast by a tree onto adjacent canopy openings. Therefore, we modified the shade profile in this simulation. Given the yearly time step, daily and seasonal variation in shading by a given tree were simplified to a single snapshot that approximates the pooled yearly shading influence of that tree on the surrounding area. We used a series of concentric ovals of shade on the northern side of a trees spatial location, the size of which depended on the trees canopy radius, as an approximation (Figure 3). The pattern of shade using this approach was similar

to the pattern generated by simulations run in the software package ShadeMotion [69], which calculates the shape and spatial position of shading across an entire growing season based on inputs including tree height, canopy radius, and latitude (Figure 3).

After the shaded region is calculated for the tallest tree, the process repeats for the next-tallest tree, which may be partially shaded by the first tree (if it is nearby) and thus have mean available light <1. Available light and thus shade in patches under the second tree is then calculated according to equation 5. If the tree shades an already-shaded patch (or patches), i.e., multiple tree canopies overlap above the patch, then total shade in the patch is calculated as

$$S = S_0 + (1 - S_0) \times S_N \tag{6}$$

where S_0 is the existing shade in the patch and S_N is the shade cast by the focal tree. This process continues for all remaining sapling and mature trees in descending order of height. Oak seedlings (<1.4 m in height) do not shade each other in the model and do not have a canopy, so light available to seedlings is simply $1 - S$ where S is the total shade in the patch where the seedling is located.

As with the site quality, light availability is translated to an effect on growth using a response function *fAL* (Figure 2; note it is not bounded on [0,1]). In SOEL, there are three response functions, one for each shade tolerance category [70]. In general, more shade tolerant species do well at low light levels, but cannot translate higher light availabilities to growth as effectively as shade-intolerant species.

5.2 Growth

In the contextual forest submodel, growth is defined as an increase in dbh and growth is calculated similar to JABOWA. Briefly, a maximum or ideal growth rate for each tree is calculated and then adjusted downward based on various factors. Maximum potential growth, δD_a, in a time step is a function of the maximum height and dbh attainable for the tree species, adjusted by a factor *G* determining how early in its life the tree achieves most of its growth; increasing values of *G* mean that the tree will reach one-half its maximum dbh more quickly [27]. Actual growth

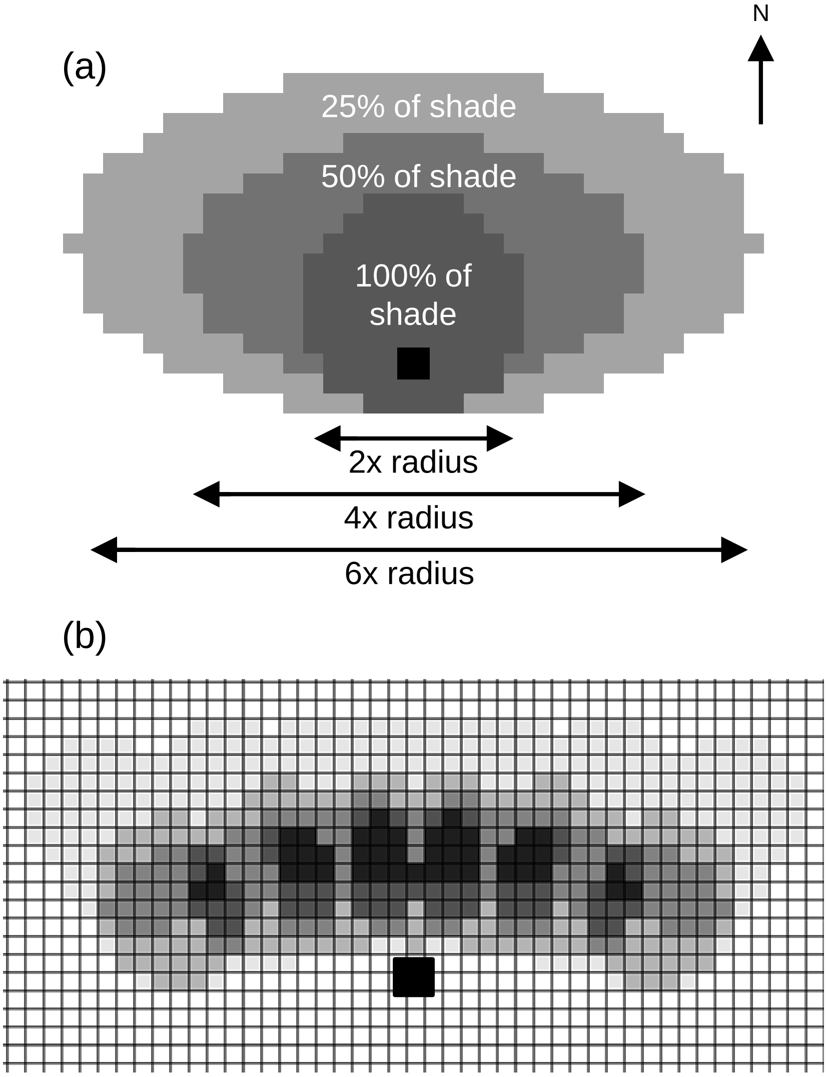

Figure 3: Comparison of simulated shade pattern across an entire growing season in the individual-based model (a) with shade pattern generated by a tree with similar height and canopy radius in the software package ShadeMotion (b). Tree location is marked in both (a) and (b) with a black square and the darker the gray, the stronger the shading effects (i.e., the area was shaded by the tree for a higher proportion of time in the growing season). The concentric elliptical approach in (a) was chosen to maximize similarity to the ShadeMotion results while minimizing complexity to facilitate faster simulations.

δD_a is calculated as the maximum growth adjusted downward by site quality *fQ*, available light *fAL* (see section 5.1), and stem count *ST* in a 3.5 m radius:

$$\delta D_a = \delta D_i \times fQ \times fAL \div ST \tag{7}$$

The stem count parameter ST is not in the original JABOWA model. It was added to this IBM to simulate the negative effects of competition by saplings for finite resources on growth, thereby preventing unrealistic stem densities and basal areas (particularly following harvesting). In this way it is analogous to the basal area limit parameter included in the original JABOWA model [27]. Several different values for the radius of the stem count (the stem density parameter d) were tested and a value of 3.5 m was chosen because it resulted in the most realistic trajectory of basal area change following forest harvest (Figure 4; [71]). Low values (*d* = 1,2) resulted in unrealistically high basal areas during the stem exclusion / understory reinitiation phases of forest succession while at higher values (d = 4,5), total stand basal area recovered to pre-harvest levels too slowly (Figure 4; [71]). Though varying *d* had a large effect on final stand basal area it did not have a strong effect on final stand breakdown by tree species (with the exception of *d* = 1) meaning that the choice of d is unlikely to have any impact on inference about final stand composition (Figure 4).

Once δD_a is calculated for a given sapling or mature tree and added to the existing dbh of the tree, the other size and shape attributes of the tree (height, basal area and leaf area index LAI) are calculated from the new dbh using allometric equations as in JABOWA [27]. The one difference in this IBM relative to JABOWA is calculation of canopy radius. In JABOWA, the canopy size of each tree was fixed as the area of the simulated cell (typically 10 $\times$ 10 m). In SOEL, the canopy is circular and changes in size as the tree grows. Canopy radius is calculated as

$$\text{Canopy Radius} = 0.385 \times \text{tree height} \div 2 \tag{8}$$

Kenefic and Nyland [72] report a range of 0.376 - 0.393 for the first value in the allometric equation. We selected 0.385 because it resulted in simulated oaks with canopy radii that most closely matched empirical our data [50].

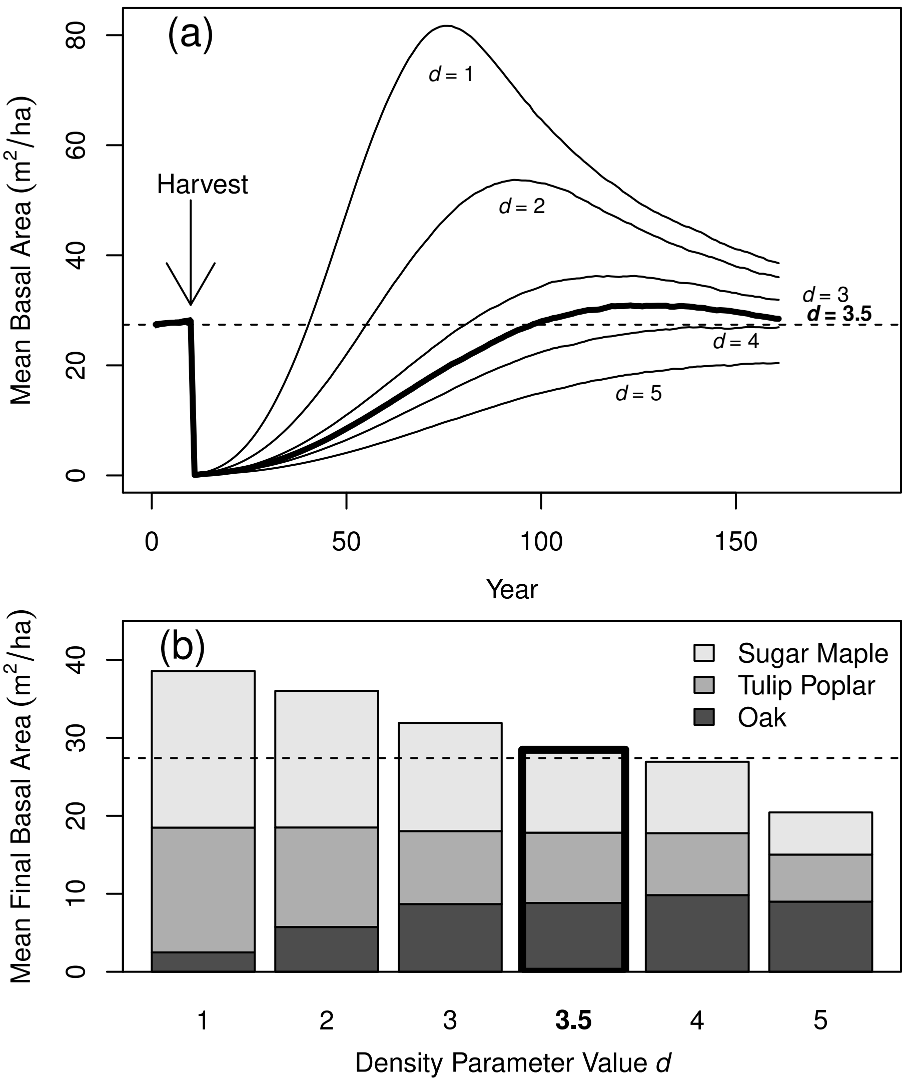

Figure 4: Comparison of (a) changes in basal area over time and (b) final basal areas for each tree species, for different values of the stem density control parameter *d*. The value of *d* used in the final model (*d* = 3.5) is highlighted in bold. The dashed lines represent pre-harvest basal area in the simulated forest stand.

5.3 Survival

Survival is directly dependent on growth, therefore reducing the number of parameters necessary to fit the model. Survival of sapling and mature trees has two components: (1) intrinsic or "background" mortality, and (2) mortality related to growth [27]. The model assumes that 2% of trees that reach a height of 1.4 m will eventually reach the maximum age for the species, given acceptable growing conditions. Therefore, there is a species-specific intrinsic background mortality of 4 / maximum age. The second component of mortality is dependent on growth. The model assumes that a tree has only a 1% chance of surviving 10 consecutive years in which no diameter growth occurs [27]. Thus, the additional probability of mortality in a given year in which diameter growth is very small or zero (<0.01 cm) can be calculated as:

$$1 - \sqrt[10]{0.01} = 0.369 \quad (9)$$

In this way, survival is tied to all the same factors (light, environmental conditions, stem density) as growth.

5.4 Reproduction

Non-oak trees (i.e., sugar maple and tulip poplar) regenerate directly as saplings. In each time step, saplings enter the simulation in a given cell with a certain probability based on site conditions and light availability [27]. The process is identical to regeneration in JABOWA except that SOEL has a smaller cell size (1 × 1 m instead of 10 × 10 m) and the sapling generation probability is adjusted accordingly. For shade-tolerant species (i.e., sugar maple), the probability ζ that a 1 × 1 m cell spawns a sugar maple sapling in a time step is:

$$\zeta = fQ \times fAL \times S_i \times 0.01 \quad (10)$$

In equation 10, *fQ* and *fAL* are values from the species-specific response functions for site quality and light in the patch, respectively, S_i is a species-specific maximum number of saplings that can be added in a 10 × 10 m area in one time step (Table 3), and 0.01 scales the probability from a 10 × 10 m area to a 1 × 1m area. For shade-intolerant species (tulip poplar), the

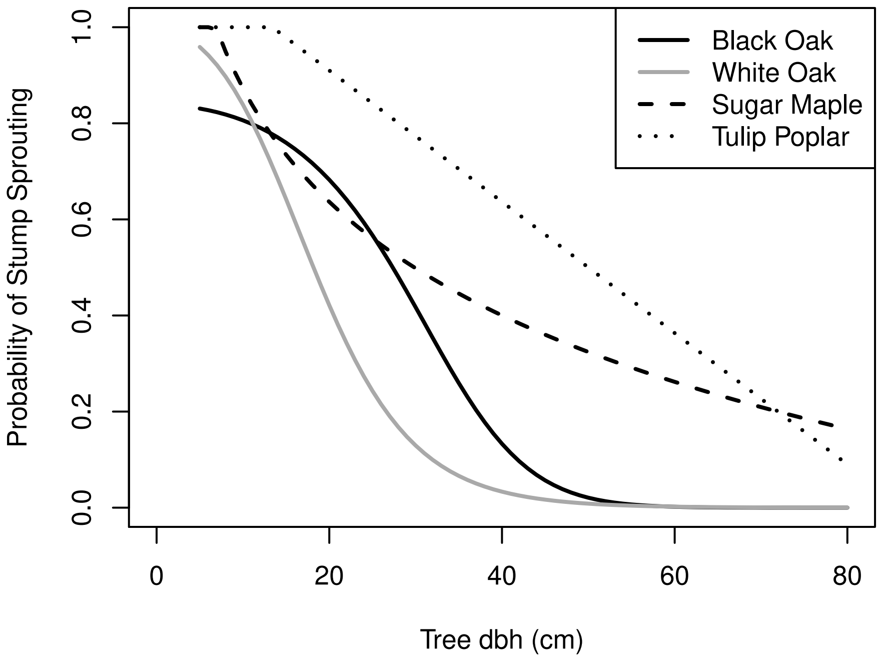

Figure 5: Probability of stump sprouting following harvest or mortality based on diameter at breast height (dbh) for the four tree species in the model. Probabilities were based on fitted regression models from the literature [73, 74, 75, 76, 77].

probability that a sapling spawns is identical to equation 10, except that when *AL* <0.99, ζ is fixed at 0 (so tulip poplar saplings can only spawn in high-light patches). Saplings enter the simulation at a random height between 1.4 and 1.67 m [27].

In JABOWA, mortality events are final and sprouting is not modeled. However, sprouting plays a key role in the successional process in eastern deciduous forests and is a key source of oak advance regeneration in particular [38]. Stump sprouting was therefore modeled explicitly in SOEL. Trees between 5 and 80 cm dbh that suffer mortality have a chance to generate a stump sprout. Probability of stump sprouting is a species-specific function of dbh and age based on logistic regression equations obtained from the literature (Figure 5; [73, 74, 75, 76, 77]). Stump sprouts enter the simulation at a random height between 1.4 and 1.67 m.

5.5 Timber Harvest

The individual-based structure of SOEL facilitates a straightforward incorporation of timber harvesting, including more complicated multiple-stage harvests. Harvests occur in simulation years defined by a rotation time. A set of rules specific to each harvesting scenario governs

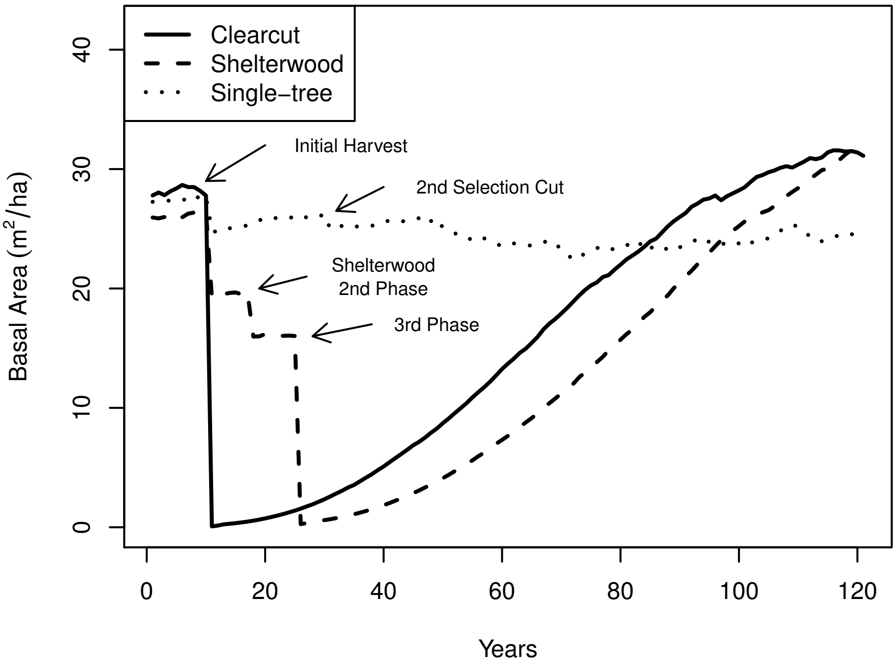

Figure 6: Changes in total stand basal area over time under three timber harvesting scenarios: clearcutting, 3-phase shelterwood harvest, and single-tree selection.

which trees will be harvested; the selected trees then die (and may resprout). The three types of timber harvest included in the model are (1) clearcutting, (2) shelterwood harvesting, and (3) single-tree selection. Harvesting rules are defined based on the harvest approach used as part of the Hardwood Ecosystem Experiment [5]. Only the core simulation area (i.e., not the buffer) is harvested.

Clearcutting is the simplest of the three scenarios. At the beginning of each rotation period (the default is a rotation of 100 years), all trees with a dbh greater than 1 cm are harvested and removed from the simulation (Figure 6). Under the shelterwood alternative, trees are harvested in three phases with a 100-year rotation. In the first phase, non-oaks in the midstory (dbh $\leq$ 25.4 cm) are removed and not allowed to resprout. In the second phase (7 years later), overstory trees are removed down to a minimum residual basal area of 16.1 m^2/ha with a preference for removing smaller, non-oak species. In the final phase (8 years after phase 2), the remaining overstory trees are harvested (Figure 6). The final harvesting approach is single-tree selection; every 20 years, trees of any species (dbh >10 cm) are removed to maintain a basal area of 25 m^2/ha in the stand with a preference for larger, more merchantable trees (Figure 6).

6 CONCLUSION

The pathway to establishment for an oak seedling is complex, involving highly variable acorn crops [50, 57], predation by insects and mammals [46, 47, 50], dispersal by small mammals [52, 78], herbivory [65], and competition and other environmental effects [66]. Oak regeneration success depends on the interaction of these and other factors (e.g., stump sprouting probability, site quality; 38). No modeling structure we are aware of has brought together all of these components into a single detailed, comprehensive model of early oak life history. To tackle this complex modeling challenge, we harnessed the power of individual-based models [26], which allowed us to track oaks (from acorns to mature trees) through time, space, and varying environmental conditions.

7 REFERENCES

[1] Swank WT, Crossley DA. Introduction and site description. In: Swank WT, Crossley DA, editors. Forest Hydrology and Ecology at Coweeta. New York, NY: Springer; 1988. p. 3–16.

[2] Franklin JF, Bledsoe CS, Callahan JT. Contributions of the long-term ecological research program. BioScience. 1990;40(7):509–523. doi:10.2307/1311319.

[3] Bierregaard RO, Lovejoy TE, Kapos V, dos Santos AA, Hutchings RW. The biological dynamics of tropical rainforest fragments. BioScience. 1992;42(11):859–866. doi:10.2307/1312085.

[4] Sheriff SL. Missouri Ozark Forest Ecosystem Project: The Experiment. In: Shifley SR, Kabrick JM, editors. Proceedings of the Second Missouri Ozark Forest Ecosystem Project Symposium: Post-treatment Results of the Landscape Experiment. Gen. Tech. Rep. NC-227. St. Paul, MN: USDA Forest Service North Central Forest Experiment Station; 2002. p. 1–25.

[5] Kalb RA, Mycroft C. The hardwood ecosystem experiment: goals, design, and implementation. In: Swihart RK, Saunders MR, Kalb RA, Haulton GS, Michler CH, editors. The Hardwood Ecosystem Experiment: a framework for studying responses to forest management. Gen. Tech. Rep. NRS-P-108. Newtown Square, PA: USDA Forest Service Northern Research Station; 2013. p. 36–59.

[6] Speer JH. Fundamentals of tree-ring research. Tucson, AZ: University of Arizona Press; 2010.

[7] Arno SF, Sneck KM. A method for determining fire history in coniferous forests. Ogden, UT: USDA Forest Service Intermountain Forest and Range Experiment Station; 1977. INT-42.

[8] Guyette RP, Dey DC, Stambaugh MC, Muzika RM. Fire scars reveal variability and dynamics of eastern fire regimes. In: Fire in Eastern Oak Forests: Delivering Science to Land Managers. Proceedings of a Conference. General Technical Report NRS-P-1. NRS-P-1. Newtown Square, PA: USDA Forest Service Northern Research Station; 2006. p. 20–39.

[9] Pitkänen Aki, Huttunen Pertti. A 1300-year forest-fire history at a site in eastern Finland based on charcoal and pollen records in laminated lake sediment. The Holocene. 1999;9(1999):311–320. doi:10.1191/095968399667329540.

[10] Betancourt JL, Van Devender TR, Martin PS. Packrat middens: the last 40,000 years of biotic change. Tucson, AZ: University of Arizona Press; 1990.

[11] Liu JG, Ashton PS. Individual based simulation models for forest succession and management. Forest Ecology and Management. 1995;73(1-3):157–175.

[12] Mladenoff DJ, Baker W. Spatial modeling of forest landscape change: approaches and applications. Cambridge, UK: Cambridge University Press; 1999.

[13] Bugmann H. A review of forest gap models. Climatic Change. 2001;51(3-4):259–305. doi:10.1023/A:1012525626267.

[14] Mladenoff DJ. LANDIS and forest landscape models. Ecological Modelling. 2004;180(1):7–19. doi:10.1016/j.ecolmodel.2004.03.016.

[15] Caswell H. Matrix population models. Sunderland, MA: Sinauer Associates; 2001.

[16] Leslie PH. On the use of matrices in certain population mathematics. Biometrika. 1945;33(3):183–212.

[17] Liang J, Picard N. Matrix model of forest dynamics: An overview and outlook. Forest Science. 2013;59(3):359–378. doi:10.5849/forsci.11-123.

[18] Zuidema PA, Jongejans E, Chien PD, During HJ, Schieving F. Integral projection models for trees: A new parameterization method and a validation of model output. Journal of Ecology. 2010;98(2):345–355. doi:10.1111/j.1365-2745.2009.01626.x.

[19] Ellner SP, Rees M. Integral projection models for species with complex demography. The American Naturalist. 2006;167(3):410–428.

[20] Enright NJ, Franco M, Silvertown J. Comparing plant life histories using elasticity analysis: the importance of life span and the number of life-cycle stages. Oecologia. 1995;104(1):79–84. doi:10.1007/BF00365565.

[21] Zuidema PA, Brienen RJW, During HJ, Guneralp B. Do persistently fast-growing juveniles contribute disproportionately to population growth? a new analysis tool for matrix models

and its application to population growth. The American Naturalist. 2009;174(5):709–719. doi:10.1086/605981.

[22] Coulson T. Integral projections models, their construction and use in posing hypotheses in ecology. Oikos. 2012;121(9):1337–1350. doi:10.1111/j.1600-0706.2012.00035.x.

[23] Jongejans E, Skarpaas O, Shea K. Dispersal, demography and spatial population models for conservation and control management. Perspectives in Plant Ecology, Evolution and Systematics. 2008;9(3-4):153–170. doi:10.1016/j.ppees.2007.09.005.

[24] Adler PB, Ellner SP, Levine JM. Coexistence of perennial plants: An embarrassment of niches. Ecology Letters. 2010;13(8):1019–1029. doi:10.1111/j.1461-0248.2010.01496.x.

[25] Jongejans E, Shea K, Skarpaas O, Kelly D, Ellner SP. Importance of individual and environmental variation for invasive species spread: a spatial integral projection model. Ecology. 2011;92(1):86–97.

[26] Grimm V, Railsback S. Individual-based modeling and ecology. Princeton, NJ: Princeton University Press; 2005.

[27] Botkin D. Forest dynamics: an ecological model. New York, NY: Oxford University Press; 1993.

[28] Shugart H, West D. Development of an Appalachian decidious forest succession model and its applicaiton to assessment of the impact of the chestnut blight. Journal of Environmental Management. 1977;5:161–179.

[29] Urban D. A versatile model to simulate forest pattern: a user's guide to ZELIG version 1.0. Charlottesville, VA: University of Virginia; 1990.

[30] Urban D, Shugart H. Individual based models of forest succession. In: Glenn-Lewin D, Peet R, Veblin T, editors. Plant Succession: Theory and Prediction. London, UK: Chapman and Hall; 1992. p. 249–292.

[31] Seagle SW, Liang SY. Application of a forest gap model for prediction of browsing effects on riparian forest succession. Ecological Modelling. 2001;144(2-3):213–229. doi:10.1016/S0304-3800(01)00373-8.

[32] Holm JA, Thompson JR, McShea WJ, Bourg NA. Interactive effects of chronic deer browsing and canopy gap disturbance on forest successional dynamics. Ecosphere. 2013;4(11):art144. doi:10.1890/ES13-00223.1.

[33] Pacala SW, Canham CD, Silander JA. Forest models defined by field measurements: I. The design of a northeastern forest simulator. Canadian Journal of Forest Research1. 1993;23(10):1980–1988.

[34] Bugmann HKM, Wullschleger SD, Price DT, Ogle K, Clark DF, Solomon AM. Comparing the performance of forest gap models in North America. Climatic Change. 2001;51(3-4):349–388. doi:10.1023/A:1012537914881.

[35] Fralish JS. The Central Hardwood Forest: its boundaries and physiographic provinces. St. Paul, MN: U.S. Department of Agriculture, Forest Service, North Central Forest Experiment Station; 2003. NC-GTR-234.

[36] McShea WJ. The influence of acorn crops on annual variation in rodent and bird populations. Ecology. 2000;81(1):228–238. doi:10.2307/177146.

[37] McShea W, Schwede G. Variable acorn crops: responses of white-tailed deer and other mast consumers. Journal of Mammalogy. 1993;74(4):999–1006. doi:Doi 10.2307/1382439.

[38] Johnson PS, Shifley SR, Rogers R. The ecology and silviculture of oaks. Cambridge, MA: CABI International; 2009.

[39] Aldrich P, Parker G, Romero-Severson J. Confirmation of oak recruitment failure in Indiana old-growth forest: 75 years of data. Forest Science. 2005;51:406–416.

[40] Nowacki G, Abrams MD. The demise of fire and "mesophication" of forests in the eastern United States. BioScience. 2008;58(2):123–138. doi:10.1641/b580207.

[41] Abrams MD. Fire and the development of oak forests. BioScience. 1992;42(5):346–353.

[42] Dey D. The ecological basis for oak silviculture in eastern North America. In: McShea WJ, Healy WM, editors. Oak Forest Ecosystems. Baltimore, MD: Johns Hopkins University Press; 2002. p. 60–79.

[43] Morrissey RC, Jacobs DF, Seifert JR, Fischer BC, Kershaw Ja. Competitive success of natural oak regeneration in clearcuts during the stem exclusion stage. Canadian Journal of Forest Research. 2008;38(6):1419–1430. doi:10.1139/X08-018.

[44] Swaim JT, Dey DC, Saunders MR, Weigel DR, Thornton CD, Kabrick JM, et al. Predicting the height growth of oak species (Quercus) reproduction over a 23-year period following clearcutting. Forest Ecology and Management. 2016;364:101–112. doi:10.1016/j.foreco.2016.01.005.

[45] McShea WJ, Healy WM, Devers P, Fearer T, Koch FH, Stauffer D, et al. Forestry matters: decline of oaks will impact wildlife in hardwood forests. Journal of Wildlife Management. 2007;71(5):1717–1728. doi:10.2193/2006-169.

[46] Bellocq MI, Jones C, Dey DC, Turgeon JJ. Does the shelterwood method to regenerate oak forests affect acorn production and predation? Forest Ecology and Management. 2005;205(1-3):311–323. doi:10.1016/j.foreco.2004.10.013.

[47] Lombardo JA, McCarthy BC. Silvicultural treatment effects on oak seed production and predation by acorn weevils in southeastern Ohio. Forest Ecology and Management. 2008;255(7):2566–2576. doi:10.1016/j.foreco.2008.01.017.

[48] Miller BF, Campbell TA, Laseter BR, Ford WM, Miller KV. White-tailed deer herbivory and timber harvesting rates: Implications for regeneration success. Forest Ecology and Management. 2009;258(7):1067–1072. doi:10.1016/j.foreco.2009.05.025.

[49] Crimmins SM, Edwards JW, Ford WM, Keyser PD, Crum JM. Browsing patterns of white-tailed deer following increased timber harvest and a decline in population density. International Journal of Forestry Research. 2010;2010:1–7. doi:10.1155/2010/592034.

[50] Kellner KF, Riegel JK, Swihart RK. Effects of silvicultural disturbance on acorn infestation and removal. New Forests. 2014;45(2):265–281. doi:10.1007/s11056-014-9409-9.

[51] Kellner KF. Interactive effects of animals and silviculture on the early life history of oak [Ph.D. Dissertation]. Purdue University; 2015.

[52] Kellner KF, Lichti NI, Swihart RK. Midstory removal reduces effectiveness of oak (Quercus) acorn dispersal by small mammals in the Central Hardwood Forest region. Forest Ecology and Management. 2016;375:182–190. doi:10.1016/j.foreco.2016.05.042.

[53] Grimm V, Berger U, Deangelis DL, Polhill JG, Giske J, Railsback SF. The ODD protocol: a review and first update. Ecological Modelling. 2010;221:2760–2768. doi:10.1016/j.ecolmodel.2010.08.019.

[54] Wilensky U. NetLogo. Evanston, IL: Northwestern University Center for Connected Learning and Computer-Based Modeling; 1999.

[55] Jenkins MA, Parker GR. Composition and diversity of woody vegetation in silvicultural openings of southern Indiana forests. Forest Ecology and Management. 1998;109(1-3):57–74. doi:10.1016/S0378-1127(98)00256-4.

[56] Saunders M, Arseneault J. Pre-treatment analysis of woody vegetation composition and structure on the hardwood ecosystem experiment research units. In: Swihart RK, Saunders MR, Kalb RA, Haulton GS, Michler CH, editors. The Hardwood Ecosystem Experiment: a framework for studying responses to forest management. Gen. Tech. Rep. NRS-P-108. Newtown Square, PA: USDA Forest Service Northern Research Station; 2013. p. 96–125.

[57] Lusk JJ, Swihart RK, Goheen JR. Correlates of interspecific synchrony and interannual variation in seed production by deciduous trees. Forest Ecology and Management. 2007;242(2-3):656–670. doi:10.1016/j.foreco.2007.01.084.

[58] Downs Aa, McQuilkin WE. Seed production of southern Appalachian oaks. Journal of Forestry. 1944;42:913–920.

[59] Gibson LP. Insects that damage white oak acorns. Upper Darby, PA: USDA Forest Service Northeastern Forest Experiment Station; 1972.

[60] Vander Wall SB, Kuhn KM, Beck MJ. Seed removal, seed predation, and secondary dispersal. Ecology. 2005;86(3):801–806.

[61] Moore JE, Mceuen AB, Swihart RK, Contreras Ta, Steele Ma. Determinants of seed removal distance by scatter-hoarding rodents in deciduous forests. Ecology. 2007;88(10):2529–2540. doi:10.1890/07-0247.1.

[62] Steele MA, HadjChikh LZ, Hazeltine J. Caching and feeding decisions by *Sciurus carolinensis*: Responses to weevil-infested acorns. Journal of Mammalogy. 1996;77(2):305–314. doi:10.2307/1382802.

[63] Haas JP, Heske EJ. Experimental Study of the Effects of Mammalian Acorn Predators on Red Oak Acorn Survival and Germination. Journal of Mammalogy. 2005;86(5):1015–1021. doi:10.1644/1545-1542(2005)86[1015:ESOTEO]2.0.CO;2.

[64] Lombardo JA, McCarthy BC. Seed germination and seedling vigor of weevil-damaged acorns of red oak. Canadian Journal of Forest Research. 2009;39(8):1600–1605. doi:10.1139/X09-079.

[65] Kellner KF, Swihart RK. Herbivory on planted oak seedlings across a habitat edge created by timber harvest. Plant Ecology. 2017;218:213–223. doi:10.1007/s11258-016-0678-6.

[66] Kellner KF, Swihart RK. Timber harvest and drought interact to impact oak seedling growth and survival in the Central Hardwood Forest. Ecosphere. 2016;7(10):e01473. doi:10.1002/ecs2.1473.

[67] Whittaker J, Whitehead C, Somers M. The neglog transformation and quantile regression for the analysis of a large credit scoring database. Applied Statistics. 2005;54(5):863–878. doi:10.1111/j.1467-9876.2005.00520.x.

[68] Nowak DJ. Estimating leaf area and leaf biomass of open-grown deciduous urban trees. Forest Science. 1996;42(4):504–507.

[69] Quesada F, Somarriba E, Malek M, Andrews K. ShadeMotion 2.2: simulation of tree shades in horizontal or tilted plots. Turrialba, Costa Rica: CATIE; 2010.

[70] Bonan G. A simulation analysis of environmental factors and ecological processes in North American boreal forests. In: Shugart H, Leemans R, Bonan G, editors. A Systems Analysis of the Global Boreal Forest. Cambridge, UK: Cambridge University Press; 1992. p. 404–427.

[71] Oliver CD, Larson BC. Forest stand dynamics. New York, NY: Wiley; 1996.

[72] Kenefic LS, Nyland RD. Sugar Maple Height-Diameter and Age-Diameter Relationships in an Uneven-Aged Northern Hardwood Stand. Northern Journal of Applied Forestry. 1999;16(1):43–47.

[73] True R. Studies on sprout reproduction of yellowpoplar as related to decay. Morgantown, WV: West Virginia University Agricultural Experiment Station; 1953.

[74] Wendel G. Stump sprout growth and quality of several Appalachian hardwood species after clearcutting. Upper Darby, PA: USDA Forest Service Northeastern Forest Experiment Station; 1975.

[75] Beck D, Della-Bianca L. Yellow-poplar: characteristics and management. USDA Forest Service Agriculture Handbook No. 583; 1981.

[76] MacDonald J, Powell G. Relationships between stump sprouting and parent-tree diameter in sugar maple in the 1st year following clear-cutting. Canadian Journal of Forest Research. 1983;13(3):390–394.

[77] Weigel DR, Peng CYJ. Predicting stump sprouting and competitive success of five oak species in southern Indiana. Canadian Journal of Forest Research. 2002;32(4):703–712. doi:10.1139/x02-042.

[78] Moore JE, Swihart RK. Importance of fragmentation-tolerant species as seed dispersers in disturbed landscapes. Oecologia. 2007;151(4):663–674. doi:10.1007/s00442-006-0616-8.
