# Supplementary material for: Simulation of oak early life history and interactions with disturbance via an individual-based model, SOEL
Source: PLoS One. 2017 Jun 20;12(6):e0179643. doi: 10.1371/journal.pone.0179643 (PMC5478140; doi:10.1371/journal.pone.0179643)
Supplement: S2 Code — (ZIP) [file pone.0179643.s003.zip › S2_Regression_Models.pdf]

S2 Code: Regression Models

Kellner, KF and Swihart, RK. 2017. Simulation of oak early life history and interactions with disturbance via an individual-based model, SOEL. PLOS ONE.

Table A. Description of candidate variables included in the candidate set for regression models of early oak life history parameters (Table B).

Candidate Variable	Type	Description
Species	Categorical	Oak species; white oak = 1
Harvest	Categorical	Location relative to midstory removal/shelterwood harvest; inside harvest = 1
Weevil	Categorical	Was acorn infested with weevils?
Mast	Continuous	Index of ambient acorn production in a given year
Dist	Continuous	Distance acorn was dispersed (m)
Cached	Categorical	Was acorn cached in the soil?
Ht	Continuous	Height of oak seedling (cm)
Ht2	Continuous	Squared height of oak seedling (to identify potential quadratic effect of height)
Shade	Continuous	Canopy cover above seedling; ranges from 0 (fully open) to 1 (fully closed)
Browse	Categorical	Was seedling browsed in the previous time step?
Age	Continous	Age (in years) of seedling
randInd	Random Effect	Standard deviation of random effect of individual tree or seedling
randYr	Random Effect	SD of random effect of year
randObs	Random Effect	SD of random effect of observation (i.e., residual SD)

Table B. Summary of fitted regression models for early oak life history parameters. R and JAGS code used to fit the models are found in the associated files.

Parameter	Source	Candidate Variables	Regression Type	Final Model	Code Files
meanAcorn	[1]	*Species; Harvest; randInd; randYr*	Exponential	Yearly sequence of observed values at HEE; or 2.00 + *randYr*(0, 1.13)	parameter_acorn_production.R
pDispersal	[1]	*Species; Harvest; Weevil; Mast; randYr*	Logistic	0.52 + 0.16 × *Species* + 0.13 × *Harvest* – 2.00 × *Weevil* + *randYr*(0, 0.63)	parameter_acorn_dispersal.R
pUndispEaten	[2]	*Species; Harvest; Mast; randYr*	Logistic	-0.18 + 1.47 × *Species* + 1.61 × *Harvest* + 1.21 × *Mast*	parameters_acorn_fate.R
dispDist	[2]	*Species; Harvest; Mast; randYr*	Weibull	2.07 – 0.115 × *Harvest* + 0.114 × *Mast*	parameters_acorn_fate.R
pCache	[2]	*Species; Harvest; Mast; Dist; randYr*	Logistic	-2.50 + 0.09 × *Dist* + *randYr*(0, 1.66)	parameters_acorn_fate.R
pDispEaten	[2]	*Species; Harvest; Mast; Cached; randYr*	Logistic	1.50 + 0.58 × *Harvest* + 1.14 × *Mast* – 5.42 × *Cached*	parameters_acorn_fate.R
pBrowse	[3]	*Species; Harvest; Ht;* Ht^2*; randYr*	Logistic	-5.49 + 0.30 × *Species* + 0.18 * *Ht* – 0.0017 × Ht^2 + *randYr*(0, 0.32)	parameter_seedling_herbivory.R
pSurv	[4]	*Species; Ht; Shade; Browse; Age; randInd; randYr*	Logistic	All years: -0.60 + 0.10 × *Species* + 0.37 × *Shade* + 0.58 × *Age*<br>No drought: 2.60 - 0.73 × *Shade*<br>Drought: -0.53 + 0.20 × *Species* + 0.53 × *Shade* + 0.44 × *Age*	parameter_seedling_survival.R
meanGr	[4]	*Species; Ht; Shade; Browse; randInd; randObs; randYr*	Normal (growth inverse-logit transformed)	All years: 1.47 + 0.33 × *Species* - 0.88 × *Shade* – 0.88 × *Browse* + *randInd*(0, 0.12) + *randObs*(0, 1.29)<br>No drought: 2.10 + 0.46 × *Species* - 1.74 × *Shade* – 0.98 × *Browse* + *randInd*(0, 0.20) + *randObs*(0, 1.47)<br>Drought: 1.07 + 0.17 × *Species* - 0.33 × *Shade* – 0.74 × *Browse* + *randInd*(0, 0.13) + *randObs*(0, 1.04)	parameter_seedling_growth.R
pGerm	[5,6]	*Weevil; Cached*	N/A; based on prior studies	*Cached* = 0.77<br>*Not Cached* = 0.09<br>*Not Cached & Weeviled* = 0.02	N/A

Literature Cited

1. Kellner KF, Riegel JK, Swihart RK. Effects of silvicultural disturbance on acorn infestation and removal. New For. 2014;45: 265–281. doi:10.1007/s11056-014-9409-9
2. Kellner KF, Lichti NI, Swihart RK. Midstory removal reduces effectiveness of oak (Quercus) acorn dispersal by small mammals in the Central Hardwood Forest region. For Ecol Manage. 2016;375: 182–190.
3. Kellner KF, Swihart RK. Oak seedling herbivory across a habitat edge created by timber harvest. Plant Ecol. 2016. doi:10.1007/s11258-016-0678-6
4. Kellner KF, Swihart RK. Timber harvest and drought interact to impact oak seedling growth and survival in the Central Hardwood Forest. Ecosphere. 2016;7: e01473. doi:10.1002/ecs2.1473
5. Haas JP, Heske EJ. Experimental Study of the Effects of Mammalian Acorn Predators on Red Oak Acorn Survival and Germination. J Mammal. 2005;86: 1015–1021. doi:10.1644/1545-1542(2005)86[1015:ESOTEO]2.0.CO;2
6. Lombardo JA, McCarthy BC. Seed germination and seedling vigor of weevil-damaged acorns of red oak. Can J For Res. 2009;39: 1600–1605. doi:10.1139/X09-079
